# SLC26A4 Targeted to the Endolymphatic Sac Rescues Hearing and Balance in *Slc26a4* Mutant Mice

**DOI:** 10.1371/journal.pgen.1003641

**Published:** 2013-07-11

**Authors:** Xiangming Li, Joel D. Sanneman, Donald G. Harbidge, Fei Zhou, Taku Ito, Raoul Nelson, Nicolas Picard, Régine Chambrey, Dominique Eladari, Tracy Miesner, Andrew J. Griffith, Daniel C. Marcus, Philine Wangemann

**Affiliations:** 1 Anatomy & Physiology Department, Kansas State University, Manhattan, Kansas, United States of America; 2 Otolaryngology Branch, National Institute on Deafness and Other Communication Disorders, National Institutes of Health, Rockville, Maryland, United States of America; 3 Department of Pediatrics, Division of Nephrology, School of Medicine, University of Utah, Salt Lake City, Utah, United States of America; 4 Inserm, UMRS 970, Centre de recherche PARCC (Paris centre de recherche cardiovasculaire); Faculté de Médecine Paris Descartes, Sorbonne Paris Cité, Paris, France; 5 Département de Physiologie, HEGP, AP-HP, Paris, France; 6 Comparative Medicine Group, Kansas State University, Manhattan, Kansas, United States of America; King's College London, United Kingdom

## Abstract

Mutations of *SLC26A4* are a common cause of human hearing loss associated with enlargement of the vestibular aqueduct. *SLC26A4* encodes pendrin, an anion exchanger expressed in a variety of epithelial cells in the cochlea, the vestibular labyrinth and the endolymphatic sac. *Slc26a4* ^Δ/Δ^ mice are devoid of pendrin and develop a severe enlargement of the membranous labyrinth, fail to acquire hearing and balance, and thereby provide a model for the human phenotype. Here, we generated a transgenic mouse line that expresses human *SLC26A4* controlled by the promoter of *ATP6V1B1*. Crossing this transgene into the *Slc26a4* ^Δ/Δ^ line restored protein expression of pendrin in the endolymphatic sac without inducing detectable expression in the cochlea or the vestibular sensory organs. The transgene prevented abnormal enlargement of the membranous labyrinth, restored a normal endocochlear potential, normal pH gradients between endolymph and perilymph in the cochlea, normal otoconia formation in the vestibular labyrinth and normal sensory functions of hearing and balance. Our study demonstrates that restoration of pendrin to the endolymphatic sac is sufficient to restore normal inner ear function. This finding in conjunction with our previous report that pendrin expression is required for embryonic development but not for the maintenance of hearing opens the prospect that a spatially and temporally limited therapy will restore normal hearing in human patients carrying a variety of mutations of *SLC26A4*.

## Author Summary

Mutations of *SLC26A4* are the most common cause for hearing loss associated with a swelling of the inner ear. This human disease is largely recapitulated in a mutant mouse model. Mutant mice lack *Slc26a4* expression and their inner ears swell during embryonic development, which leads to failure of the cochlea and the vestibular organs resulting in deafness and loss of balance. *SLC26A4* is normally found in the cochlea and vestibular organs of the inner ear as well as in the endolymphatic sac, which is a non-sensory part of the inner ear. The multitude of sites where *SLC26A4* is located made the goal to restore function through restoration look futile, unless some sites were more important than others. Here, we generated a new mutant mouse that expresses *SLC26A4* in the endolymphatic sac but not in the cochlea or the vestibular organs of the inner ear. Fantastically, this mouse did not develop the detrimental swelling of the inner ear and even more exciting, the mouse developed normal hearing and balance. Our study provides the proof-of-concept that a therapy aimed at repairing the endolymphatic sac during embryonic development is sufficient to restore a life-time of normal hearing and balance.

## Introduction

Enlargement of the vestibular aqueduct (EVA; OMIM #600791) is a malformation of the temporal bone that is commonly observed in children with sensorineural hearing loss [1], [2], [3], [4], [5]. Mutations of *SLC26A4* are the most common cause for EVA-associated hearing loss that can either be non-syndromic (*DFNB4*; OMIM # 600791) or syndromic with enlargement of the thyroid gland (Pendred syndrome; OMIM #274600). *SLC26A4* codes for the anion exchanger pendrin that transports anions such as Cl^−^, I^−^ and HCO_3_^−^ [6], [7]. Although EVA is a malformation of the temporal bone, it is not the cause for hearing loss since no correlation was found between the degree of EVA and the severity of hearing impairment [8]. EVA, however, is an indication of an enlargement of the endolymphatic duct epithelium that was present during embryonic development. Cartilage cells that form in the periphery of the endolymphatic duct epithelium preserve the diameter of the duct in a ‘fossil-like’ record when they give rise to the bone of the vestibular aqueduct.

**Figure 1:**
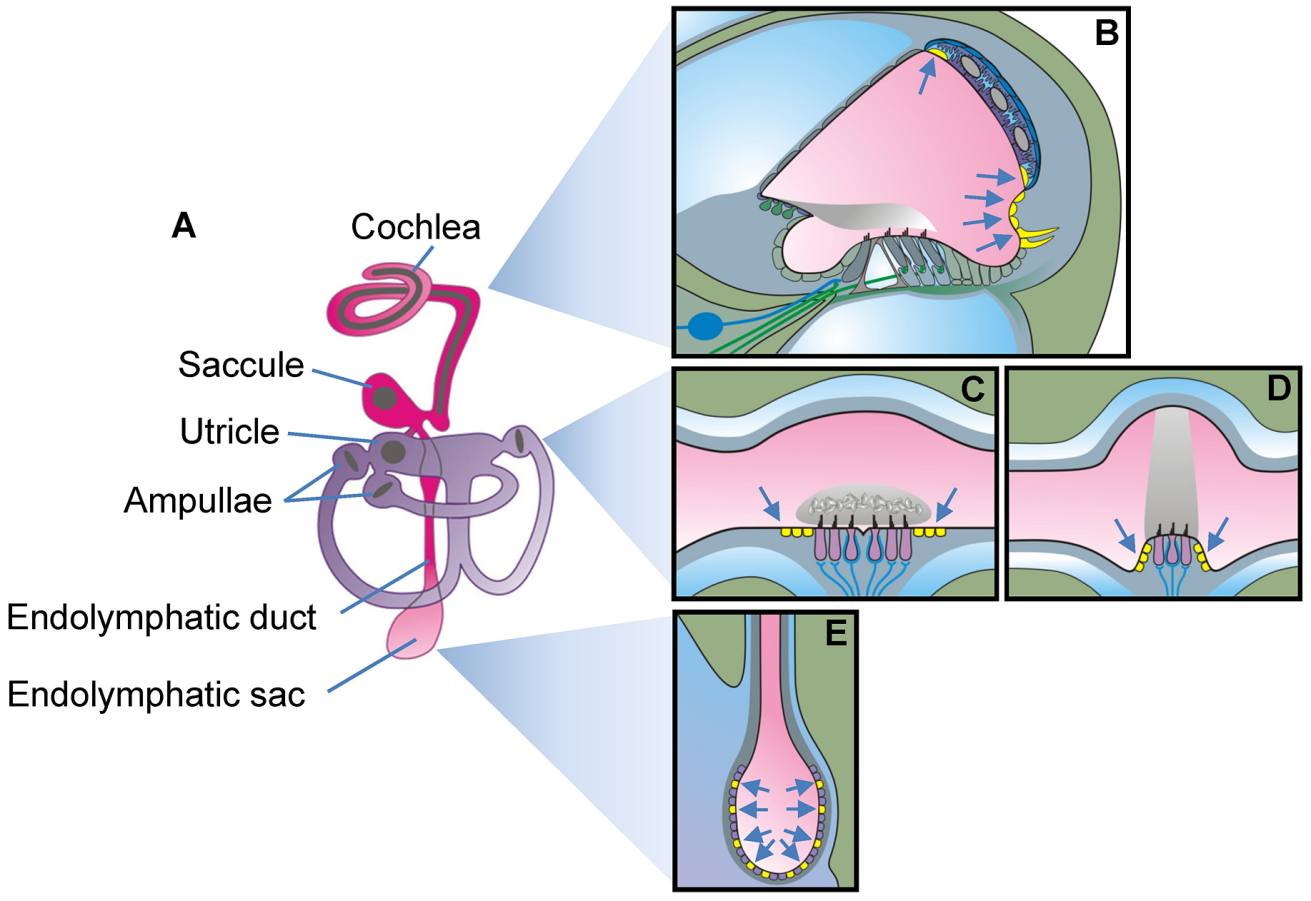
Schematic diagram of the inner ear. **A**) Diagram of the membranous labyrinth. The two continuous luminal fluid spaces of the mature inner ear are filled with endolymph (*pink* and *purple*). **B–E**) Diagrams of a cross section of one cochlear turn (**B**), of the utricle or saccule (**C**), of one ampulla (**D**) and the endolymphatic sac (**E**). Cells that express pendrin (*yellow cells* pointed to by *arrows*) are diagrammed in mature tissues.

The mature inner ear consists of seven interconnected fluid spaces that house six sensory organs (Fig. 1): The cochlea for hearing, the utricle and saccule for sensing linear acceleration including gravity, and three ampullae with semicircular canals for sensing angular acceleration in three spatial axes. The seventh fluid compartment is the endolymphatic duct and sac, which is devoid of sensory cells and which is suspected to play a role in fluid homeostasis [9], [10]. Pendrin is expressed in a variety of epithelial cells that enclose endolymph, which is the luminal fluid of the inner ear (Fig. 1). Pendrin is expressed in outer sulcus, spiral prominence and spindle-shaped cells in the cochlea, transitional cells in the utricle, saccule and ampullae and mitochondria-rich cells (synonym: Forkhead-related or FORE cells) of the endolymphatic sac [11], [12]. Each cell type represents a small domain in the heterogeneous epithelium that encloses endolymph. The many locations and cell types that express pendrin in a normal inner ear made the goal to restore function through restoration of expression look futile unless some sites of expression were more important than others.

The earliest onset of pendrin expression in the murine inner ear occurs in the endolymphatic sac at embryonic day E11.5, which precedes the onset of expression in the cochlea by 3 days, in the saccule and utricle by 4 days, and in the ampullae by 5 days [13]. The expression in the endolymphatic sac surges dramatically at E14.5, a time in development when there is very little pendrin expressed elsewhere in the inner ear [13].

Studies in a mouse model, *Slc26a4^Δ/Δ^*, have revealed that loss of pendrin leads to an enlargement of endolymph volume followed by an acidification and a failure to develop normal hearing and balance [13], [14]. The onset of the enlargement in the cochlea and the endolymphatic sac occurs at E14.5 which precedes the onset of the luminal acidification by 1 day in the cochlea and by 3 days in the endolymphatic sac [13]. The enlargement develops in *Slc26a4^Δ/Δ^* mice between E14.5 and E18.5, which is the phase of rapid growth of the cochlea [4]. The coincidence of the surge in pendrin expression in the endolymphatic sac at E14.5 and the onset of the enlargement in *Slc26a4^Δ/Δ^* mice points to the importance of pendrin expression in the endolymphatic sac for inner ear fluid homeostasis.

We hypothesized that restoration of pendrin expression in the endolymphatic sac would prevent enlargement and permit normal development of the cochlea and the vestibular labyrinth including the acquisition of sensory function. To test this hypothesis, we generated a mouse line that expresses human pendrin *SLC26A4* controlled by the promoter of the B1-subunit of the human vacuolar H^+^ ATPase (*ATP6V1B1*) and crossed this transgene into the *Slc26a4^Δ/Δ^* line to generate mice that lack expression of mouse pendrin but express human pendrin in the endolymphatic sac. No expression of pendrin protein was detected in these mice in the cochlea or the vestibular labyrinth but in mitochondria-rich cells of the endolymphatic sac. Analysis of this mouse model revealed normal hearing and balance function. Our data indicate that the expression of pendrin solely in the endolymphatic sac of the inner ear is sufficient to permit the development of normal hearing and balance.

## Results

### Generation of Tg(+);Slc26a4^Δ/Δ^ transgenic mice

A transgenic mouse line, referred to as Tg*(B1-hPDS)* ^Tg/+^; *Slc26a4^+/+^* and abbreviated here to Tg(+);*Slc26a4^+/+^*, was created by the laboratory of Dr. Dominique Eladari (Paris, France) [15]. This mouse expresses human *SLC26A4* (formerly named *hPDS*) controlled by the promoter of *ATP6V1B1*, which codes for the B1-subunit of the vH^+^ATPase. Transgenic founders were crossed with wild-type C57BL/6× CBA F1 mice and three Tg(+);*Slc26a4^+/+^* mice were shipped to Kansas State University (Manhattan, Kansas, USA). At Kansas State University, Tg(+);*Slc26a4^+/+^* mice were crossed with *Slc26a4^Δ/Δ^* mice, which are maintained in an isogenic 129S6SvEv background, to generate the desired Tg(+);*Slc26a4* ^Δ/Δ^ mice in an F2 generation. Expression of *SLC26A4* (human pendrin) in this mouse was expected to originate solely from the transgene since Exon 8 in the *Slc26a4* ^Δ^ allele was replaced with a neomycin-cassette that introduced a frame-shift [14]. Littermates with the genotype Tg(−);*Slc26a4* ^Δ/Δ^ served as negative controls. These mice were expected to lack functional pendrin protein expression. Further, littermates with genotypes Tg(−);*Slc26a4* ^Δ/+^, Tg(+);*Slc26a4* ^Δ/+^, and Tg(+);*Slc26a4* ^+/+^ served as positive controls. These mice were expected to express murine pendrin with or without augmentation of human pendrin and have normal hearing and balance.

### mRNA expression

**Figure 2:**
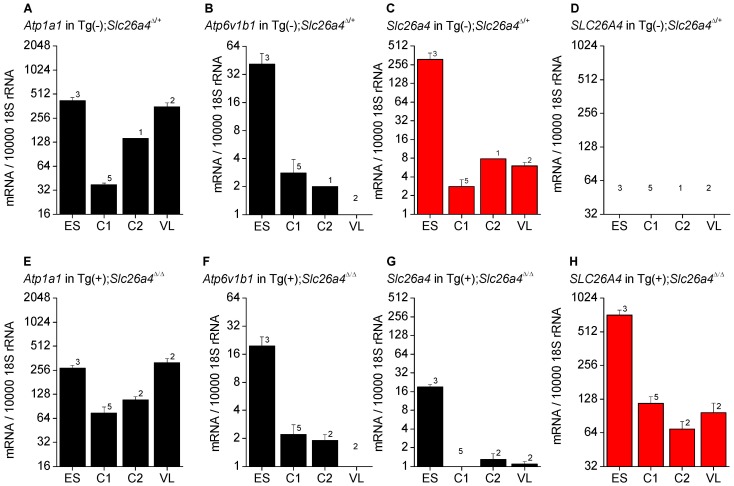
*Atp1a1*, *Atp6v1b1*, *Slc26a4*, and *SLC26A4* mRNA levels in inner ear tissues. Expression was determined by quantitative RT-PCR performed on total RNA. Total RNA was isolated from microdissected tissues obtained from Tg(−);*Slc26a4* ^Δ/+^ (**A–D**) and Tg(+);*Slc26a4* ^Δ/Δ^ mice (**E–H**). Endolymphatic sacs (ES) were isolated from mice at age E17.5. Cochleae were isolated at ages E17.5 (C1) and P2 (C2). Vestibular labyrinths (VL), consisted of saccule, utricle, ampullae and semicircular canals without endolymphatic sacs, were isolated at age P8. The expression of endogenous mouse *Atp1a1*, *Atp6v1b1*, and *Slc26a4*, and of transgenic human *SLC26A4* mRNA was normalized to the expression of 18S rRNA. Note that the expression pattern of human *SLC26A4* in Tg(+);*Slc26a4* ^Δ/Δ^ mice resembles the pattern of mouse *Slc26a4* in Tg(−);*Slc26a4* ^Δ/+^ mice (both patterns *highlighted in red*). Numbers inside graphs represent the number of experiments.

Expression of *Atp1a1*, *Atp6v1b1*, *Slc26a4* and *SLC26A4* was determined by quantitative RT-PCR and normalized to the expression of 18S rRNA (Fig. 2). The highest levels of *Atp6v1b1* and *Slc26a4* mRNA among the different inner ear tissues were found in the endolymphatic sac (Fig. 2B and C). Expression of *Slc26a4* was reduced by factors between 6 and 16 in Tg(+);*Slc26a4* ^Δ/Δ^ mice compared to Tg(−);*Slc26a4* ^Δ/+^ mice (Fig. 2C vs G). Expression levels of *Atp1a1 and Atp6v1b1* exhibited a similar pattern among inner ear tissues of Tg(−);*Slc26a4* ^Δ/+^ and Tg(+);*Slc26a4* ^Δ/Δ^ mice (Fig. 2A vs E and Fig. 2B vs F). Most interesting, expression levels of human *SLC26A4* in Tg(+);*Slc26a4* ^Δ/Δ^ resembled the pattern of mouse *Slc26a4* in Tg(−);*Slc26a4* ^Δ/+^ mice with the highest levels being expressed in the endolymphatic sac (Fig. 2C vs H). Whether or not expression levels of human *SLC26A4* in Tg(+);*Slc26a4* ^Δ/Δ^ exceeded expression levels of mouse *Slc26a4* in Tg(−);*Slc26a4* ^Δ/+^ mice remained undetermined, since the efficiency of the reverse transcription of mRNA into cDNA remains generally unknown in quantitative RT-PCR experiments. Taken together, the data demonstrate that the transgene restored pendrin mRNA expression to the endolymphatic sac, the cochlea and the vestibular labyrinth of the inner ear.

### Protein expression - eGFP expression

**Figure 3:**
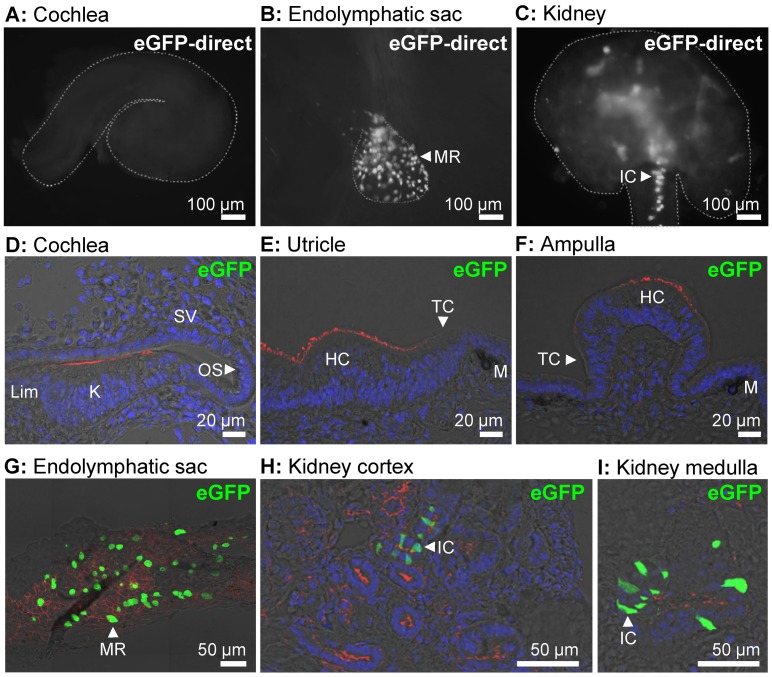
Transgene-encoded eGFP expression. **A–C**) Expression of eGFP was monitored as direct fluorescence in cochlear ducts (**A**), endolymphatic sacs (**B**), and kidney slices (**C**) freshly isolated from E15.5 Tg(*B1-eGFP*) mice. The outline of the imaged tissues is marked (*dashed-line*). **D–I**) Expression of eGFP was evaluated by immunocytochemistry in the cochlea (**D**), utricle (**E**), ampulla (**F**), endolymphatic sac (**G**), kidney cortex (**H**), and kidney medulla (**I**) from E15.5 Tg(*B1-eGFP*) mice. Staining consisted of immunocytochemistry of eGFP (*green*), F-actin (*red*) and nucleic acids (*blue*). The number of mice represented by these images are 2 for images **A–C** and 2 for images **D–I**. MR, mitochondria-rich cells; IC, intercalated cells; Lim, spiral limbus; K, Kölliker's organ; OS, outer sulcus; SV, stria vascularis; HC, vestibular hair cells; TC, transitional cells; M, melanocytes.

The ability of the *ATP6V1B1* promoter to drive protein expression in different tissues including the cochlea, the vestibular labyrinth and the endolymphatic sac was evaluated in a transgenic mouse line, Tg(*B1-eGFP*) in which the expression of eGFP is controlled by the same 6.9 kb promoter of the human *ATP6V1B1* gene that drives the expression of human pendrin in Tg(+);*Slc26a4* ^Δ/Δ^ mice [16]. No expression of eGFP was detected in the cochlea or the vestibular labyrinth of E15.5 Tg(*B1-eGFP*) mice, although expression was present in the endolymphatic sac and the kidney (Fig. 3). These data suggest that the *ATP6V1B1* promoter does not drive protein expression in the cochlea or the vestibular labyrinth.

### Protein expression - Western blotting

**Figure 4:**
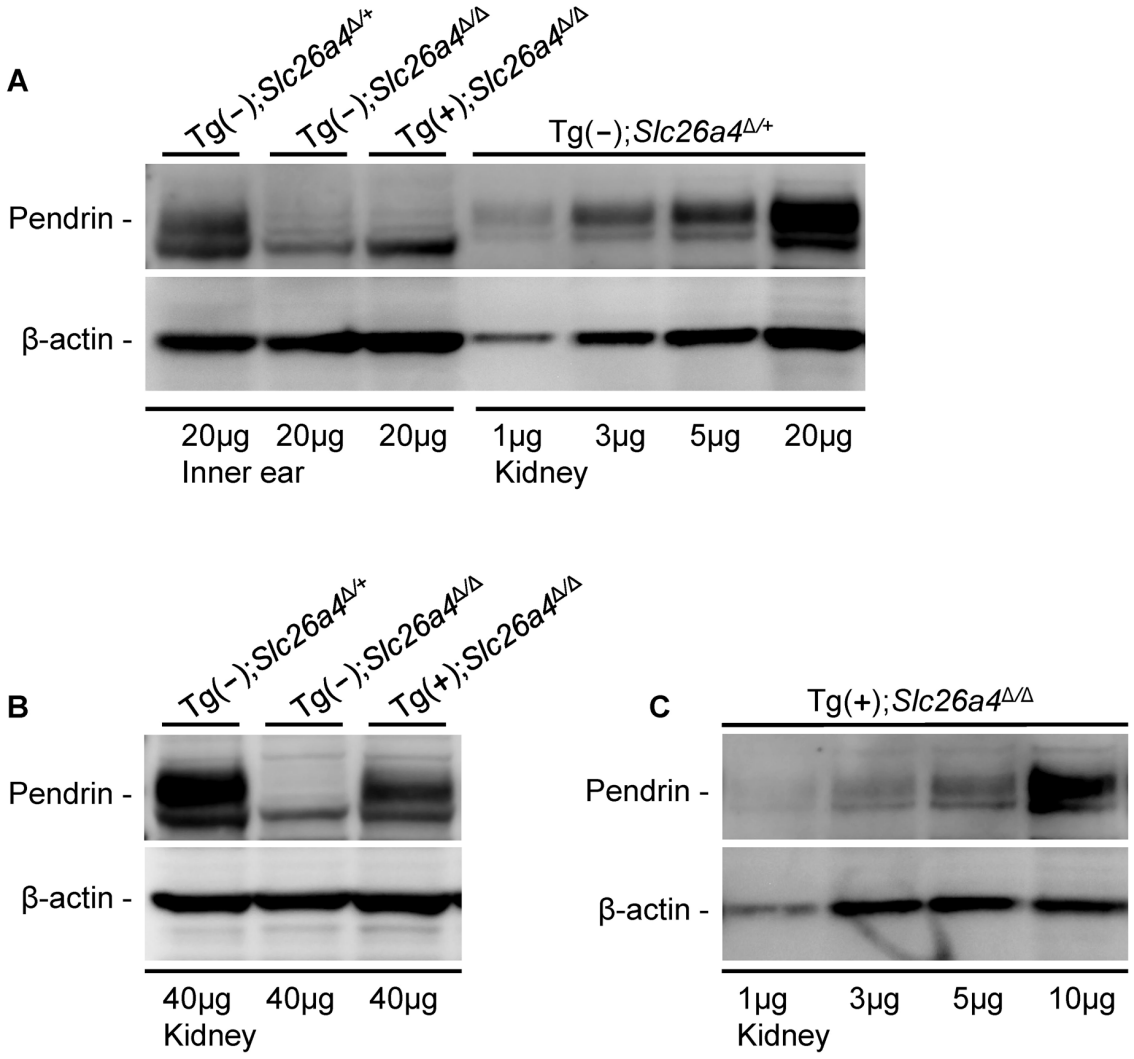
Pendrin expression evaluated by Western blotting. Proteins in crude membranes prepared from pooled inner ear tissues consisting of soft tissues from the cochlea and the vestibular labyrinth devoid of endolymphatic sac and crude membranes prepared from kidneys of Tg(−)*Slc26a4* ^Δ/+^, Tg(−)*Slc26a4* ^Δ/Δ^ and Tg(+)*Slc26a4* ^Δ/Δ^ mice were resolved by gel-electrophoresis and detected with an anti-pendrin antibody (Pds #1) and an anti-β-actin antibody. β-actin served as a loading control. **A**) Comparison of pendrin expression in membranes prepared from inner ears of Tg(−)*Slc26a4* ^Δ/+^, Tg(−)*Slc26a4* ^Δ/Δ^ and Tg(+)*Slc26a4* ^Δ/Δ^ mice to expression levels in descending amounts of membranes prepared from kidneys of Tg(−)*Slc26a4* ^Δ/+^ mice. **B**) Comparison of pendrin expression in membranes prepared from kidneys of Tg(−)*Slc26a4* ^Δ/+^, Tg(−)*Slc26a4* ^Δ/Δ^ and Tg(+)*Slc26a4* ^Δ/Δ^ mice. **C**) Expression levels of pendrin in descending amounts of membranes prepared from kidneys of Tg(+)*Slc26a4* ^Δ/Δ^ mice. Data shown in **A**, **B** and **C** are each representative of 2 biological replicates.

Soft tissues of the cochlea and the vestibular labyrinth, exclusive of the endolymph sac, were collected from adult mice by microdissection and pooled into an ‘inner ear’ sample. Crude membrane protein preparations were obtained from these inner ear samples and from kidneys and subjected to gel-electrophoresis and Western blotting. Membrane proteins were obtained from Tg(+);*Slc26a4* ^Δ/Δ^ mice as well as from Tg(−);*Slc26a4* ^Δ/+^ mice, which served as positive controls, and from Tg(−);*Slc26a4* ^Δ/Δ^ mice, which served as negative controls. Pendrin was detected in the inner ear and kidney of Tg(−);*Slc26a4* ^Δ/+^ mice as a ∼110 kDa band (Fig. 4A). Inner ear from Tg(+);*Slc26a4* ^Δ/Δ^ mice lacked this band. The observation that there was no difference in the pattern of faint bands between inner ears from Tg(+);*Slc26a4* ^Δ/Δ^ mice and Tg(−);*Slc26a4* ^Δ/Δ^ mice, which is the negative control, suggests that pendrin was either not detectable or not present. The pendrin band, however, was found in kidney from Tg(+);*Slc26a4* ^Δ/Δ^ mice (Fig. 4B), which suggests that the antibody recognizes both mouse and human pendrin. The observation that pendrin was detected at similar levels in descending amounts of kidney proteins isolated from Tg(−);*Slc26a4* ^Δ/+^ mice (Fig. 4A) and Tg(+);*Slc26a4* ^Δ/Δ^ mice (Fig. 4C) suggests that the detection threshold for mouse and human pendrin was similar. Whether the antibody differed in the sensitivity between mouse and human pendrin remains unknown, since the relative abundance of mouse pendrin in kidneys of Tg(−);*Slc26a4* ^Δ/+^ mice and human pendrin in kidneys of Tg(+);*Slc26a4* ^Δ/Δ^ mice is not known.

The meaning of pendrin being not detectable in the inner ear was evaluated by comparison of the intensity of the pendrin band in inner ear to the intensities in descending amounts of kidney protein (Fig. 4A). This comparison suggests that a ∼5-fold lower amount pendrin should have been detectable in the inner ear. This means that pendrin expression in the inner ear of Tg(+);*Slc26a4* ^Δ/Δ^ mice is either absent or expressed at a level that does not exceed 20% of the expression level in Tg(−);*Slc26a4* ^Δ/+^ mice.

### Paint-fill of the cochlea and the endolymphatic sac

**Figure 5:**
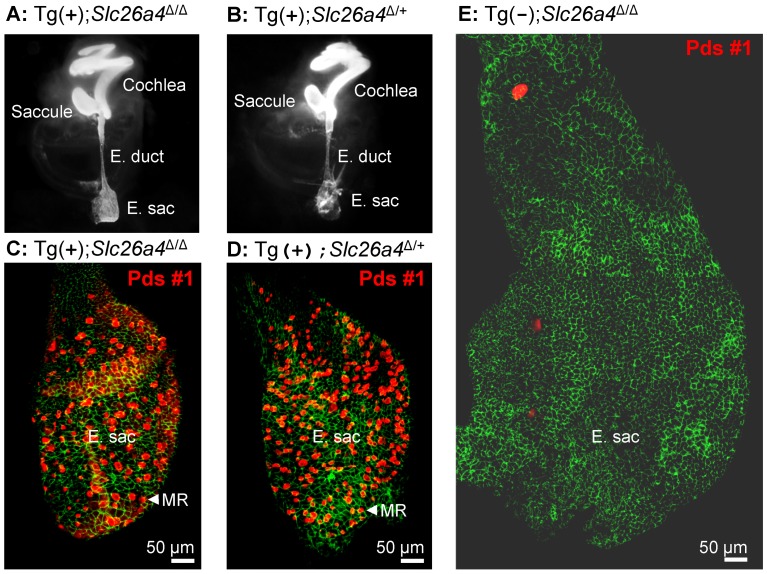
Histology and pendrin expression in the embryonic endolymphatic sac. **A–B**) Paint filled inner ears from Tg(+);*Slc26a4* ^Δ/Δ^ and Tg(+);*Slc26a4* ^Δ/+^ mice at age E15.5. Note that neither the endolymphatic duct (E. duct) nor the endolymphatic sac (E. sac) were enlarged. **C–E**) Pendrin protein expression in the endolymphatic sac at age E16.5. Staining consisted of immunocytochemistry of pendrin (*red*) and F-actin (*green*). Images provide comparison of whole mounts from Tg(+);*Slc26a4* ^Δ/Δ^ mice (**C**), Tg(+);*Slc26a4* ^Δ/+^ mice (**D**), and Tg(−);*Slc26a4* ^Δ/Δ^ mice (**E**). Note that images **C–E** are presented at the same scale. The number of mice represented by these images is 2 for **A**, 2 for **B**, 3 for **C**, 4 for **D**, and 1 for **E**. MR, mitochondria-rich cells.

Temporal bones from Tg(+);*Slc26a4* ^Δ/+^ and Tg(+);*Slc26a4* ^Δ/Δ^ mice were isolated at E15.5, fixed and injected with white paint (Fig. 5A and B). Most striking is that there was no enlargement of the endolymphatic sac, duct or cochlea in Tg(+);*Slc26a4* ^Δ/Δ^ mice and that the morphology of Tg(+);*Slc26a4* ^Δ/+^ and Tg(+);*Slc26a4* ^Δ/Δ^ mice was grossly similar. These data demonstrate that the introduction of the transgene rescued the malformation previously described in *Slc26a4* ^Δ/Δ^ mice [10], [14].

### Histology and pendrin expression in the endolymphatic sac

Whole-mounted specimens of the endolymphatic sac were prepared for immunocytochemistry from Tg(+);*Slc26a4* ^Δ/Δ^, Tg(−);*Slc26a4* ^Δ/Δ^, and Tg(+);*Slc26a4* ^Δ/+^ mice (Fig. 5C–E). Most striking is the enlargement and lack of pendrin expression in the endolymphatic sac of Tg(−);*Slc26a4* ^Δ/Δ^ mice (Fig. 5E) and the similarity in size and similarity in pendrin expression between the endolymphatic sac of Tg(+);*Slc26a4* ^Δ/Δ^ mice (Fig. 5C) and Tg(+);*Slc26a4* ^Δ/+^ mice (Fig. 5D). These data demonstrate that the transgene drives pendrin expression in the endolymphatic sac and that the introduction of the transgene rescued the malformation [10], [14].

### Gross morphology of the cochlea

**Figure 6:**
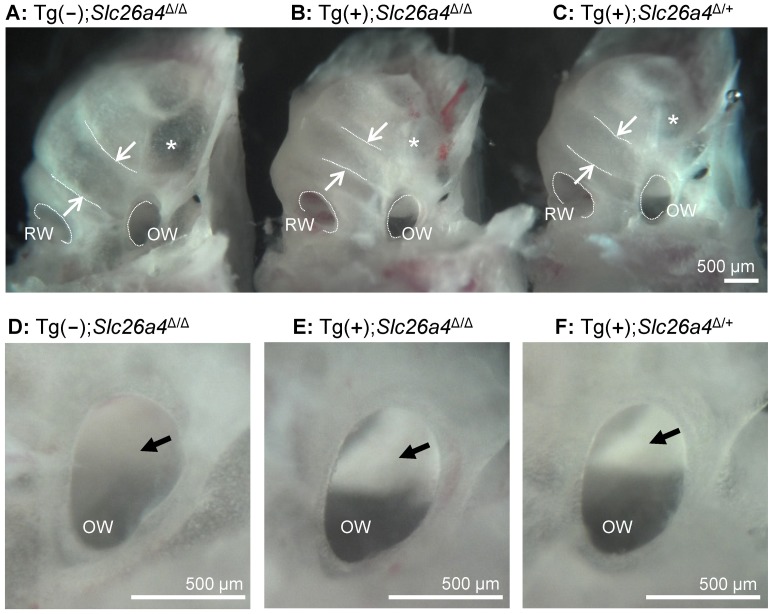
Gross morphology of the cochlea isolated by microdissection. **A–C**) Overview images of cochleae from a Tg(−);*Slc26a4* ^Δ/Δ^, Tg(+);*Slc26a4* ^Δ/Δ^ and Tg(+);*Slc26a4* ^Δ/+^ mice. The width of the lower cochlear turn is marked with arrows and the round (RW) and oval window (OW) are labeled. A region with apparently thinner bone is marked (*). **D–F**) Enlarged view of the oval window. Normal otoconia in the saccule that reflect the light and appear bright white were seen in Tg(+);*Slc26a4* ^Δ/Δ^ and Tg(+);*Slc26a4* ^Δ/+^ mice but not in Tg(−);*Slc26a4* ^Δ/Δ^ (*arrow*). The number of mice represented by these images is 1 for **A** and **D**, and 3 pairs for images **B** & **C** and **E** & **F**.

Gross morphological examination of inner ears revealed greater similarity between Tg(+);*Slc26a4* ^Δ/Δ^ mice and Tg(+);*Slc26a4* ^Δ/+^ than between Tg(+);*Slc26a4* ^Δ/Δ^ mice and Tg(−);*Slc26a4* ^Δ/Δ^ mice (Fig. 6A–C). Cochlear turns in Tg(+);*Slc26a4* ^Δ/Δ^ mice appeared normal in width and did not show widening of turns or thinning of the otic capsule that was seen in Tg(−);*Slc26a4* ^Δ/Δ^ and that was previously described in *Slc26a4* ^Δ/Δ^ mice [12], [17]. Inspection of the oval window revealed ‘glittering’ otoconia in the saccule in Tg(+);*Slc26a4* ^Δ/Δ^ and Tg(+);*Slc26a4* ^Δ/+^ mice in contrast to Tg(−);*Slc26a4* ^Δ/Δ^ mice where no ‘glittering’ was visible (Fig. 6D–F).

### Histology and pendrin expression in the cochlea

**Figure 7:**
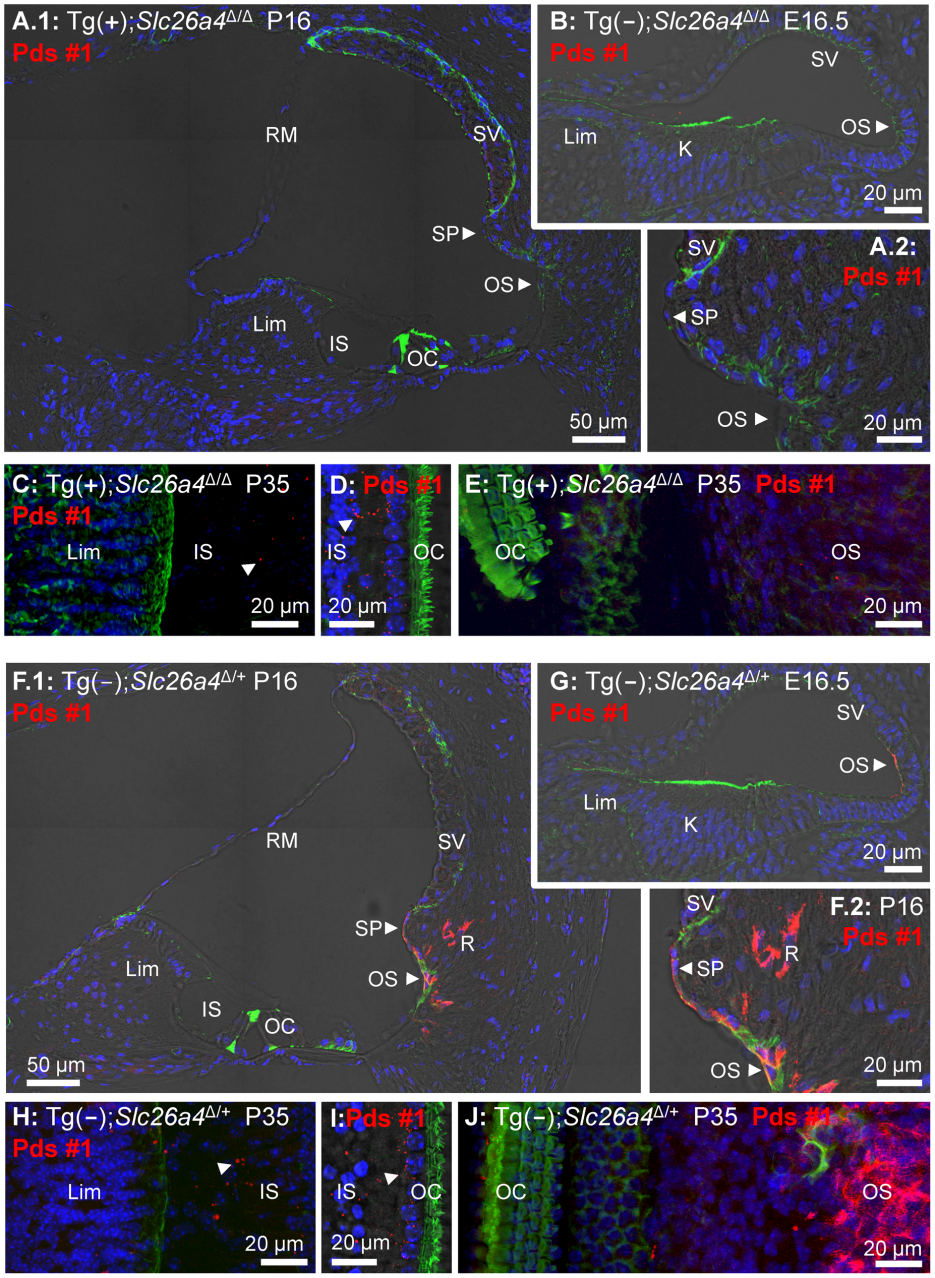
Histology and pendrin expression in the cochlea. Staining in all images consisted of immunocytochemistry of pendrin (Pds #1 antibody; *red*), F-actin (*green*) and nucleic acids (*blue*). Images provide comparison of cryosections (**A–B**) and whole-mounted specimens (**C–E**) from Tg(+);*Slc26a4* ^Δ/Δ^ mice to cryosections (**F–G**) and whole-mounted specimens (**H–J**) from Tg(−);*Slc26a4* ^Δ/+^ mice. Whole-mounted specimens in **C**, **E**, **H** and **J** were imaged by detecting fluorescence in 25 optical sections that were recorded in 1 µm intervals and projected into a single plane. Whole-mounted specimens in **D** and **I** were imaged by detecting fluorescence in 8 optical sections that were recorded in 1 µm intervals, projected into a single plane and overlaid onto a single brightfield image. The number of pairs of mice represented by these images are 2 for image **A** & **F**, 3 for **B** & **G**, and 1 for **C–E** & **H–J** with 3 sections being evaluated per animal. K, Kölliker's organ; OS, outer sulcus; Lim, spiral limbus; IS, inner sulcus; OC, organ of Corti; SP, spiral prominence; SV, stria vascularis; RM, Reissner's membrane. Additional images using an alternative anti-pendrin antibody (Pds #2) and alternative positive controls (Tg(+);*Slc26a4* ^Δ/+^ mice) are provided in the Supplement (Fig. S1).

Midmodiolar sections of cochlear tissues were prepared for immunocytochemistry from Tg(+);*Slc26a4* ^Δ/Δ^ mice and positive controls consisting of Tg(−);*Slc26a4* ^Δ/+^ or Tg(+);*Slc26a4* ^Δ/+^ mice. No evidence for cochlear enlargement was found in Tg(+);*Slc26a4* ^Δ/Δ^ mice at E16.5 (Fig. 7B), P1 (Fig. S1A), P16 (Fig. 7A; Fig. S1E) or P18 (Fig. S1C) suggesting that the introduction of the transgene rescued the cochlear malformation previously described in *Slc26a4* ^Δ/Δ^ mice, which includes a ∼10-fold enlargement of the cochlea [12], [14]. No detectable pendrin expression was found in the spiral prominence or outer sulcus epithelium of the cochlea in Tg(+);*Slc26a4* ^Δ/Δ^ mice although prominent expression was observed in these cells in positive controls (Fig. 7 and S1). The absence of pendrin in Tg(+);*Slc26a4* ^Δ/Δ^ mice was observed with two different anti-pendrin antibodies (Pds #1 and Pds #2). The patterns of pendrin expression in the positive controls, Tg(−);*Slc26a4* ^Δ/+^ and Tg(+);*Slc26a4* ^Δ/+^ mice, were similar for both antibodies to the pattern previously observed in *Slc26a4* ^Δ/+^ mice [11], [12], [13]. Expression of pendrin was further examined in whole-mounted specimens that encompassed the spiral limbus, organ of Corti and outer sulcus. No detectable pendrin expression was found at age P35 in the spiral limbus of Tg(+);*Slc26a4* ^Δ/Δ^ mice (Fig. 7C) or Tg(−);*Slc26a4* ^Δ/+^ mice (Fig. 7H) in contrast to the prominent expression of pendrin in the outer sulcus epithelia of Tg(−);*Slc26a4* ^Δ/+^ mice (Fig. 7J). For completeness, it needs to be reported that some punctate staining was found in nerve terminals near inner hair cells of Tg(+);*Slc26a4* ^Δ/Δ^ (Fig. 7D) and Tg(−);*Slc26a4* ^Δ/+^ mice (Fig. 7I).

### Endocochlear potential and pH

**Figure 8:**
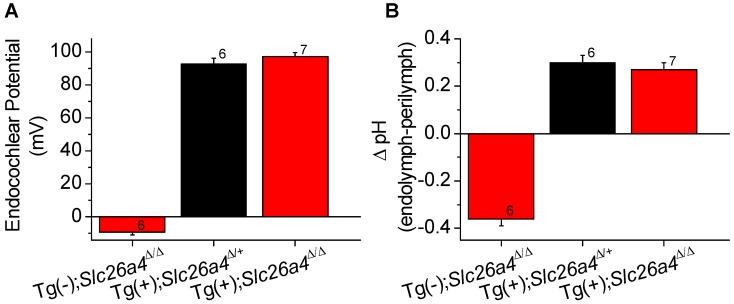
Endocochlear potential and pH. Endocochlear potential (**A**) and the difference between endolymphatic and perilymphatic pH (**B**) were measured in Tg(−);*Slc26a4* ^Δ/Δ^, Tg(+);*Slc26a4* ^Δ/+^, and Tg(+);*Slc26a4* ^Δ/Δ^ mice. Numbers near the error bars represent the number of experiments.

The endocochlear potential and the difference in pH between endolymph and perilymph was measured with double-barreled ion selective electrodes in Tg(−);*Slc26a4* ^Δ/Δ^ mice, Tg(+);*Slc26a4* ^Δ/+^ mice, and Tg(+);*Slc26a4* ^Δ/Δ^ mice (Fig. 8). Tg(−);*Slc26a4* ^Δ/Δ^ mice failed to develop a normal endocochlear potential and the pH of endolymph was lower ( = more acidic) than in perilymph, as previously reported [18]. In contrast, Tg(+);*Slc26a4* ^Δ/+^ mice, as reported for *Slc26a4* ^Δ/+^ mice [18], developed a normal endocochlear potential and a normal endolymphatic pH that was higher ( = more alkaline) than in perilymph. Similar to Tg(+);*Slc26a4* ^Δ/+^ mice, Tg(+);*Slc26a4* ^Δ/Δ^ mice developed a normal endocochlear potential and a normal endolymphatic pH even though no detectable pendrin expression was observed in the cochlear epithelium. These data demonstrate that the introduction of the transgene, which rescued the malformation, also rescued the loss of the endocochlear potential and the loss of normal endolymphatic pH homeostasis.

### Hearing

**Figure 9:**
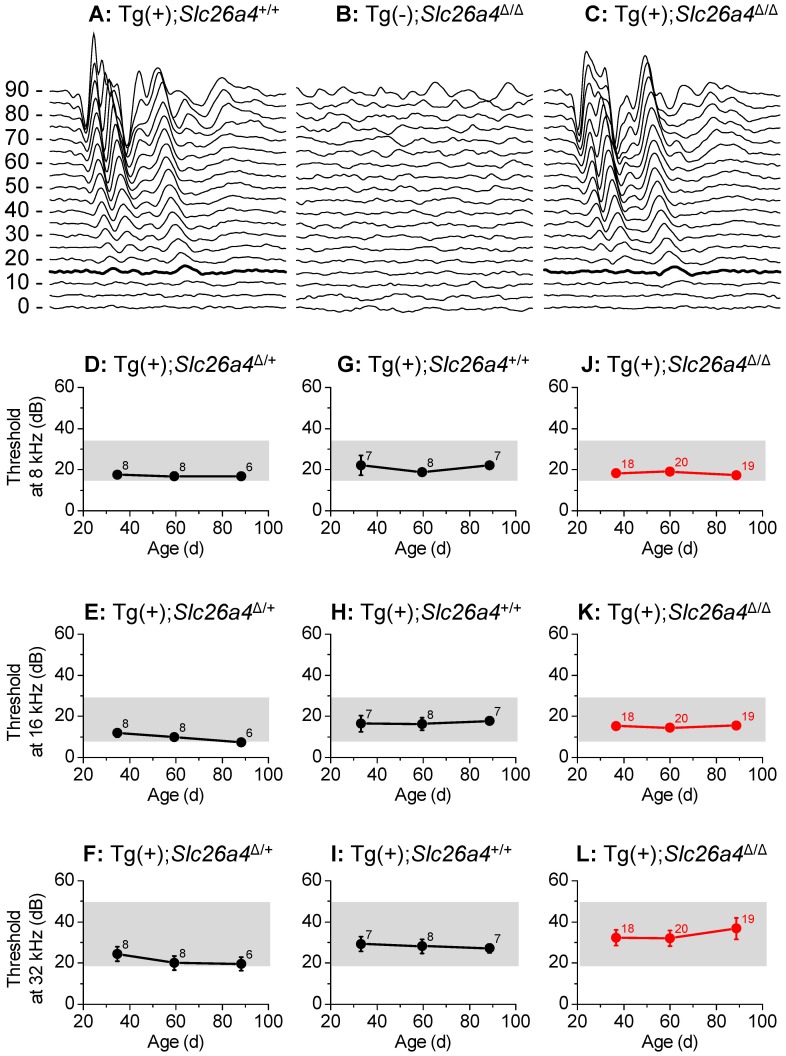
Hearing tests based on auditory brain stem responses. **A–C**) Examples of recordings of auditory brain stem responses to tone bursts of 16 kHz at amplitudes between 0 and 90 dB-SPL that were presented to Tg(+);*Slc26a4* ^+/+^ mice at P35 (**A**), Tg(−);*Slc26a4* ^Δ/Δ^ mice at P35 (**B**), and Tg(+);*Slc26a4* ^Δ/Δ^ mice at P35 (**C**). Thresholds at 15 dB-SPL are marked (*thickened trace*). **D–L**) Hearing thresholds based on auditory brain stem responses, were determined in Tg(+);*Slc26a4* ^Δ/+^ mice (**D–F**), Tg(+);*Slc26a4* ^+/+^ mice (**G–I**), and Tg(+);*Slc26a4* ^Δ/Δ^ mice (**J–L**; *highlighted in red*). Frequencies evaluated at 8 kHz (**D, G, J**), 16 kHz (**E, H, K**), and 32 kHz (**F, I, L**). The combined ranges of normal hearing in the three mouse strains (129S6, C57BL/6 and CBA) that contributed to the background of the mice are marked (*grey rectangles*). Numbers next to symbols in **D–L** represent the number of mice tested.

Hearing tests were based on auditory brain stem recordings and thresholds in response to tone bursts of 8 kHz, 16 kHz and 32 kHz. Tests performed in Tg(+);*Slc26a4* ^+/+^, Tg(−);*Slc26a4* ^Δ/Δ^ and Tg(+);*Slc26a4* ^Δ/Δ^ mice confirmed profound deafness in Tg(−);*Slc26a4* ^Δ/Δ^ mice (Fig. 9B) consistent with previous findings in *Slc26a4* ^Δ/Δ^ mice [14], [18]. Waveforms of auditory brain stem recordings as well as thresholds were similar between Tg(+);*Slc26a4* ^Δ/Δ^ mice (Fig. 9A) and Tg(+);*Slc26a4* ^Δ/+^ (Fig. 9C). These findings demonstrate that the introduction of the transgene rescued normal hearing although the cochlea did not express detectable levels of pendrin. We next evaluated whether the rescued hearing phenotype in Tg(+);*Slc26a4* ^Δ/Δ^ would be stable through at least 3 months of age. Auditory brain stem recordings were performed in Tg(+);*Slc26a4* ^Δ/+^ mice (Fig. 9D–F), in Tg(+);*Slc26a4* ^Δ/Δ^ mice (Fig. 9J–L), and in Tg(+);*Slc26a4* ^+/+^ mice (Fig. 9G–I) at 1, 2 and 3 month of age. Hearing in Tg(+);*Slc26a4* ^Δ/Δ^ mice at 8 kHz and 16 kHz was stable through 3 months. Thresholds were very similar among individuals and did not differ from Tg(+);*Slc26a4* ^Δ/+^ and Tg(+);*Slc26a4* ^+/+^ mice. A greater variability in hearing thresholds was observed at 32 kHz (Fig. 9L), with 10 of the 19 Tg(+);*Slc26a4* ^Δ/Δ^ mice maintaining excellent hearing (thresholds ≤30 dB at 32 kHz) and 5 developing a high-frequency hearing loss (thresholds ≥60 dB at 32 kHz). About one half of the Tg(+);*Slc26a4* ^Δ/Δ^ mice (9 of 19) developed progressive threshold elevations at 32 kHz with thresholds increasing by ≥10 dB between the monthly measurements. This variability is reflected in the greater error bars at 32 kHz but did not lead to a statistically significant difference between Tg(+);*Slc26a4* ^Δ/Δ^ and Tg(+);*Slc26a4* ^+/+^ mice.

### Histology and pendrin expression in the vestibular labyrinth

**Figure 10:**
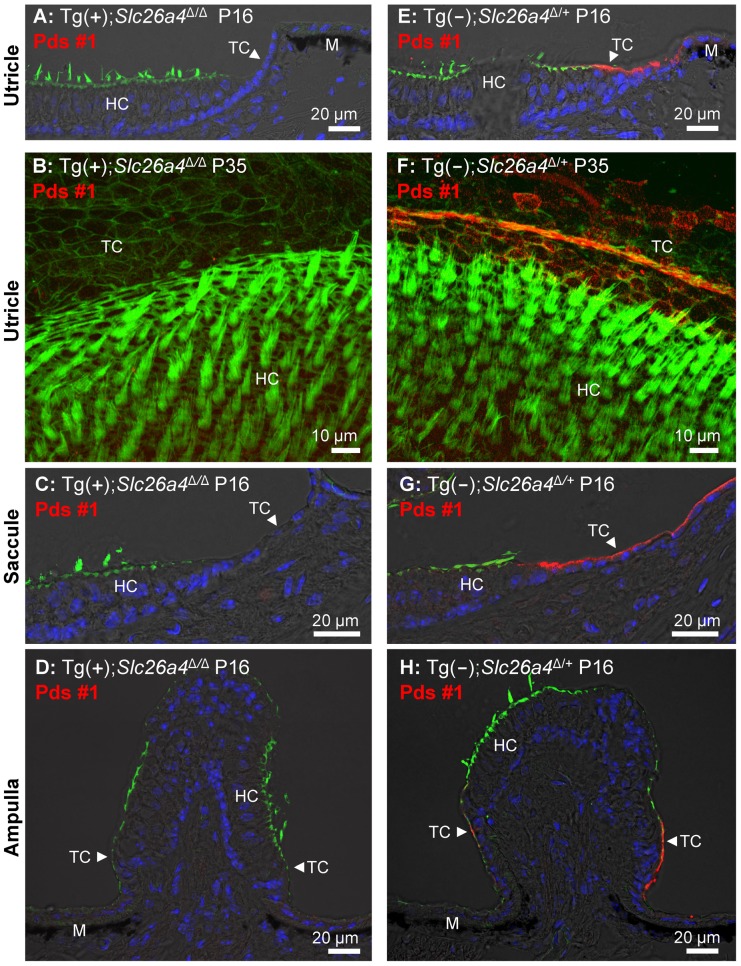
Histology and pendrin expression in the vestibular labyrinth. Staining in all images consisted of immunocytochemistry of pendrin (Pds #1 antibody; *red*) and F-actin (*green*) and of nucleic acids (*blue*), the latter with the exception of images **B** and **F**. Images provide comparison of cryosections (**A, C–D**) and whole-mounted specimens (**B**) from Tg(+);*Slc26a4* ^Δ/Δ^ to cryosections (**E, G–H**) and whole-mounted specimens (**F**) from Tg(−);*Slc26a4* ^Δ/+^ mice. Whole-mounted specimens were imaged by detecting fluorescence in 25 optical sections that were recorded in 1 µm intervals and projected into a single plane. The number of pairs of mice represented by these images are 2 for image **A** & **E**, 1 for **B** & **F**, 2 for **C** & **G**, and 2 for **D** & **H** with 3 sections being evaluated per animal. HC, vestibular hair cells; TC, transitional cells; M, melanocytes. Additional images using an alternative anti-pendrin antibody (Pds #2) and alternative positive controls (Tg(+);*Slc26a4* ^Δ/+^ mice) are provided in the Supplement (Fig. S2).

Sections and whole-mounted specimens of vestibular tissues were prepared for immunocytochemistry from Tg(+);*Slc26a4* ^Δ/Δ^ mice and positive controls consisting of Tg(−);*Slc26a4* ^Δ/+^ or Tg(+);*Slc26a4* ^Δ/+^ mice. No evidence of pendrin expression was found in the three sensory organs in Tg(+);*Slc26a4* ^Δ/Δ^ mice at P14 (Fig. S2A), P16 (Fig. 10A,C,D and Fig. S2C,G,K), P18 (S2E), and P35 (Fig. 10B). The absence of pendrin in Tg(+);*Slc26a4* ^Δ/Δ^ mice was observed with two different anti-pendrin antibodies (Pds #1 and Pds #2). In contrast, pendrin expression was found in transitional cells of the utricle, saccule and ampullae of controls that consisted of Tg(−);*Slc26a4* ^Δ/+^ or Tg(+);*Slc26a4* ^Δ/+^ mice. The expression patterns in Tg(−);*Slc26a4* ^Δ/+^ and Tg(+);*Slc26a4* ^Δ/+^ mice with both antibodies were similar to the pattern previously described in *Slc26a4* ^Δ/+^ mice [11], [12].

### Otoconia

**Figure 11:**
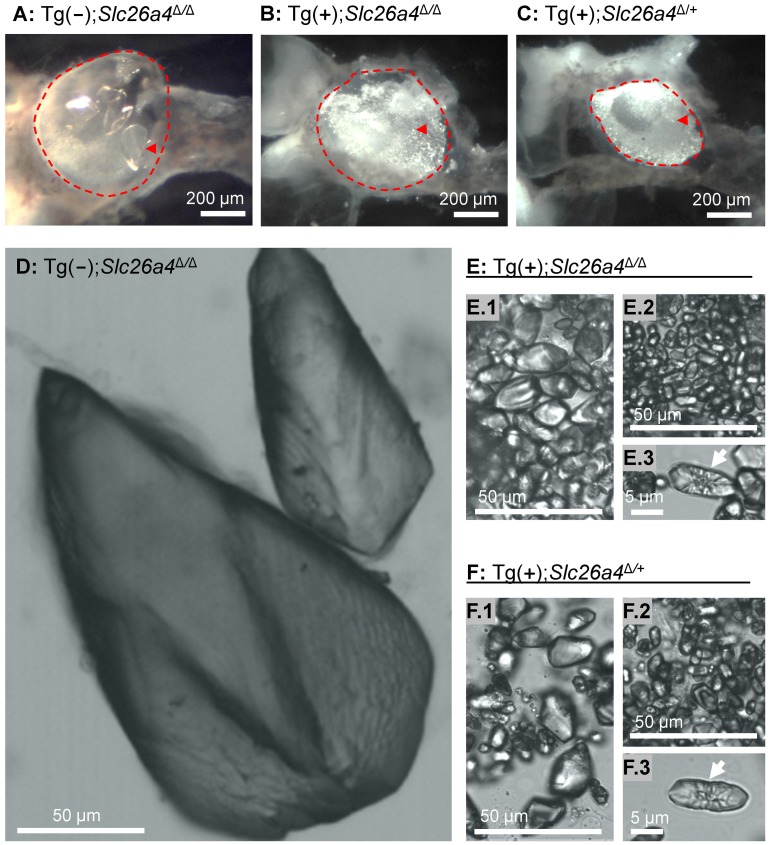
Otoconia. **A–C**) Microdissected maculae utriculi from a Tg(−);*Slc26a4* ^Δ/Δ^, Tg(+);*Slc26a4* ^Δ/Δ^ and Tg(+);*Slc26a4* ^Δ/+^ mice. The macula utriculi have been marked (*red dashed line*) and a single otoconium is indicated (*red arrow head*). **D–F**) Isolated otoconia from Tg(−);*Slc26a4* ^Δ/Δ^, Tg(+);*Slc26a4* ^Δ/Δ^ and Tg(+);*Slc26a4* ^Δ/+^ mice. Note that images **D**, **E.1**, **E.2**, **F.1** and **F.2** are presented at the same scale. Mega-otoconia were found only in Tg(−);*Slc26a4* ^Δ/Δ^ mice. Tg(+);*Slc26a4* ^Δ/Δ^ mice contained a mixture of larger and smaller otoconia, some of which showed a concentric pattern (**E.1**, **E.2** and **E.3**) that was similar to the mixture found in Tg(−);*Slc26a4* ^Δ/+^ mice (**F.1**, **F.2** and **F.3**).

Vestibular labyrinths were isolated by microdissection from Tg(−);*Slc26a4* ^Δ/Δ^, Tg(+);*Slc26a4* ^Δ/Δ^ and Tg(+);*Slc26a4* ^Δ/+^ mice and the roof of the utricle was removed to permit an unobstructed view onto the utricular macula (Fig. 11A–C). Glittering otoconia were observed in Tg(+);*Slc26a4* ^Δ/Δ^ and Tg(+);*Slc26a4* ^Δ/+^ mice and giant otoconia in Tg(−);*Slc26a4* ^Δ/Δ^. Otoconia were transferred into glass-bottom dishes and inspected by laser-scanning microscopy using a 405 nm laser. Giant otoconia from Tg(−);*Slc26a4* ^Δ/Δ^ mice were ∼10-fold larger than normal otoconia (Fig. 11D). The shape of the giant otoconia resembled the shape previously observed in *Slc26a4* ^Δ/Δ^ mice [12]. Otoconia in Tg(+);*Slc26a4* ^Δ/Δ^ and Tg(+);*Slc26a4* ^Δ/+^ mice were similar consisting in both genotypes of larger (∼20 µm, Figs. 11E.1 and F.1) and smaller (∼10 µm; Figs. 11E.2 and F.2) otoconia, some of which revealed a concentric structure (Figs. 11E.3 and F.3). These data suggest that the introduction of the transgene rescued normal otoconia formation.

### Balance

**Figure 12:**
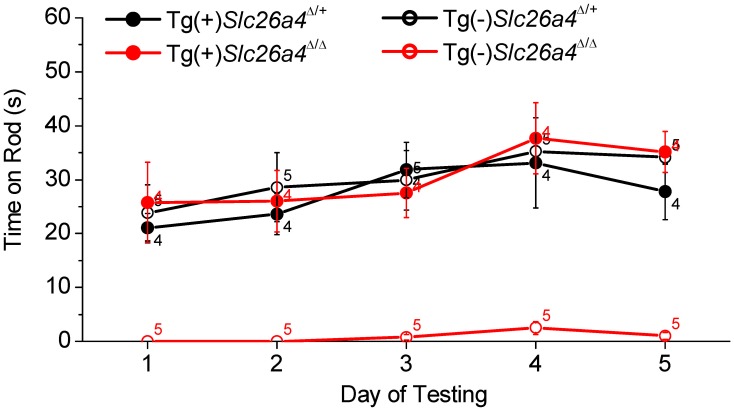
Balance tests. Tests were based on the ability of Tg(+);*Slc26a4* ^Δ/+^, Tg(+);*Slc26a4* ^Δ/Δ^, Tg(−);*Slc26a4* ^Δ/+^, Tg(−);*Slc26a4* ^Δ/Δ^ mice to balance on a rotating rod that was accelerated from 4 to 40 rpm over 60 s. Numbers near the symbols indicate the number of mice evaluated.

Balance tests were performed in Tg(+);*Slc26a4* ^Δ/Δ^ mice and Tg(+);*Slc26a4* ^Δ/+^ mice as well as in Tg(−);*Slc26a4* ^Δ/+^ and Tg(−);*Slc26a4* ^Δ/Δ^ mice (Fig. 12). Tg(−);*Slc26a4* ^Δ/Δ^ mice failed the test, which confirmed the vestibular phenotype previously described in *Slc26a4* ^Δ/Δ^ mice [14]. There was no apparent difference in the performance of Tg(+);*Slc26a4* ^Δ/Δ^, Tg(+);*Slc26a4* ^Δ/+^ and Tg(−);*Slc26a4* ^Δ/+^ mice. These data demonstrate that the introduction of the transgene rescued normal gross motor vestibular function.

## Discussion

In this study we generated a mouse that expresses human pendrin in the endolymphatic sac but lacks detectable pendrin protein expression in the cochlea or in the vestibular labyrinth. The most biologically interesting and clinically relevant observation of this study is that this mouse develops normal hearing and balance. Our findings support the hypothesis that pendrin expression in the endolymphatic sac is chiefly responsible for the development of normal endolymph volume, that lack of pendrin in the endolymphatic sac is mainly responsible for the development of the membranous labyrinth enlargement in *Slc26a4^Δ/Δ^* mice, and that the complex inner ear pathology found in *Slc26a4^Δ/Δ^* mice is largely a consequence of the enlargement during embryonic development. This hypothesis was based on the studies in *Slc26a4^Δ/Δ^* mice that revealed that the enlargement is a key event on the path toward organ failure resulting in deafness and vestibular dysfunction [12], [13], [18] and on studies in *Foxi1^−/−^* mice that lack pendrin expression in the endolymphatic sac, develop an enlargement of the inner ear, but express pendrin in the cochlea and the vestibular labyrinth [19].

To test our hypothesis, we generated a transgenic mouse, Tg(*B1-hPDS*), which expresses human *SLC26A4* (previously named *PDS*) controlled by the promoter of *ATP1V1B1* [15]. The ability of the promoter of *ATP1V1B1* to control gene expression had previously been evaluated in a transgenic mouse that expresses eGFP controlled by the promoter of *ATP6V1B1* [16], [20]. Expression of eGFP had been found in this mouse in intercalated cells of the renal collecting duct, and in narrow and clear cells of the epididymal epithelium of adult mice [16]. We found expression of eGFP in the embryonic kidney and in mitochondria-rich cells of the endolymphatic sac but not in the cochlea or the vestibular labyrinth (Fig. 3). Expression in mitochondria-rich cells of the endolymphatic sac was expected since these cells are members of the FORE family (forkhead related) of cells. FORE cells express FOXI1, which drives the expression of *Atp6v1b1* and *Slc26a4* [21], [22], [23]. Consistently, mitochondria-rich cells of the endolymphatic sac express the mRNAs *Atp6v1b1* and *Slc26a4* [24], [25] and the corresponding proteins, the B1-subunit of the vH^+^ATPase and pendrin [11], [23]. The onset of expression of *Atp6v1b1* in the endolymphatic sac is at E11.5, which is similar to the onset of pendrin [13], [26]. Thus, it was likely that the transgene Tg(*B1-hPDS*) would drive a timely expression of pendrin in mitochondria-rich cells of the endolymphatic sac.

Although FOXI1 drives the expression of *Atp6v1b1* and *Slc26a4* in FORE cells such as the mitochondria-rich cells of the endolymphatic sac, the expression of *Atp6v1b1* and *Slc26a4* is not limited to FORE cells. Indeed, *Slc26a4* is expressed in the inner ear in spiral prominence and outer sulcus epithelial cells as well as in spindle-shaped cells of the cochlea and in transitional cells of the vestibular labyrinth, none of which are FORE cells [11], [12]. Further, *Atp6v1b1* expression has been found in the spiral limbus of the cochlea, which does not contain FORE cells [25], [26]. The expression of *Atp6v1b1* in the cochlea provided the possibility that the transgene would drive an ectopic expression of pendrin in the spiral limbus. Our studies of eGFP expression (Fig. 3) and of pendrin expression by Western blotting (Fig. 4) and immunocytochemistry of whole-mounted specimens and sections using two different anti-pendrin antibodies (Pds #1 and Pds #2, Fig. 7 and S1) revealed no detectable expression in the cochlea or vestibular labyrinth of Tg(+);*Slc26a4* ^Δ/Δ^ mice. The observed absence of pendrin expression in the vestibular labyrinth of Tg(+);*Slc26a4* ^Δ/Δ^ mice (Fig. 10 and S2) is consistent with the reported lack of *Atp6v1b1* expression in the vestibular labyrinth based on detection by *in situ* hybridization [25], [26] and by quantitative RT-PCR (Fig. 2B and F), which is a more sensitive technique. The observation that human *SLC26A4* mRNA but no pendrin nor eGFP protein was detected in the cochlea or the vestibular labyrinth suggests the presence of strong translational regulation [27]. Taken together, our data demonstrate that we have generated a mouse that expresses pendrin in the endolymphatic sac but not in the cochlea or the vestibular labyrinth, although we cannot completely rule out that low levels of pendrin protein expression escaped our detection. Such low pendrin expression is unlikely the reason for the restored endolymphatic volume, since pendrin expression in the cochlea and vestibular labyrinth of *Foxi^−/−^* mice, which lack pendrin expression in the endolymphatic sac, did not prevent endolymphatic enlargement [19] and since mice that express a mutant pendrin protein that supports anion exchange at a reduced rate are deaf, develop mega-otoconia and are balance impaired [28]. Moreover, hypomorphic mutant alleles of *SLC26A4* show no difference in the resulting auditory phenotype from that of functional null alleles in patients with Pendred syndrome [29], indicating that small amounts of pendrin activity are insufficient to rescue hearing in humans.

Measurements of the endocochlear potential and pH revealed that the introduction of the transgene, which rescued normal endolymph volume, also rescued the loss of the normal endocochlear potential and the loss of the normal endolymphatic pH homeostasis (Fig. 8). It appears that the Cl^−^/HCO_3_^−^ exchanger pendrin, which is normally expressed in the apical membranes of spiral prominence and outer sulcus epithelial cells, is not the sole mechanism responsible for the alkaline pH of endolymph in a normally developed cochlea. A similar conclusion can be drawn based on measurements in the doxycycline-inducible *Slc26a4* mouse model where termination of pendrin expression at P6 led to the development of a nearly normal endocochlear potential and of a nearly normal alkaline pH [30]. We hypothesize that the epithelial barrier enclosing endolymph is permeable to H^+^, OH^−^ and HCO_3_^−^ and that the pH of endolymph follows the endocochlear potential.

Hearing and balance tests in Tg(+);*Slc26a4* ^Δ/Δ^ mice revealed normal sensory function (Fig. 9 and 12). The observation that hearing thresholds at 32 kHz had some variability in Tg(+);*Slc26a4* ^Δ/Δ^ mice and that some Tg(+);*Slc26a4* ^Δ/Δ^ mice developed progressive high-frequency hearing loss is most likely a function of the genetic background. Tg(+);*Slc26a4* ^Δ/Δ^ were generated in a F2 generation from *Slc26a4* ^Δ/Δ^ mice that were maintained isogenic in the 129S6 background and Tg(+);*Slc26a4* ^Δ/Δ^ mice that were recently generated in a mixed background of C57BL/6 and CBA. DNA from the three background strains, 129S6, C57BL/6 and CBA, which differ in their hearing thresholds, are expected to comprise variable amounts of the genomes of individual mice. Hearing thresholds for 1 to 3 month-old 129S6, C57BL/6 and CBA mice range between 20–35 dB-SPL at 8 kHz, 10–28 dB-SPL at 16 kHz and 20–50 dB-SPL at 32 Hz [18], [31], [32], [33], [34], [35]. In general, 129S6, C57BL/6 and CBA mice have similar thresholds at 8 kHz, whereas at 16 and 32 kHz CBA mice have lower thresholds than 129S6 and C57BL/6 mice. Thus, the greater variability in hearing thresholds that was observed at 32 kHz particularly in Tg(+);*Slc26a4* ^Δ/Δ^ may be due to a variability in the mixture of these background strains.

Our observation that normal hearing developed in the absence of pendrin expression in the cochlea in combination with the published finding that normal hearing was maintained when pendrin expression was terminated after completed development [30], could suggest that pendrin in the cochlea has no physiologic significance beyond the developmental phase. However, it is also conceivable that pendrin-mediated HCO_3_^−^ secretion provides a buffer that stabilizes the pH in the lateral wall tissues as well as in endolymph, and that this buffering is important during stress situations associated with normal life. Pendrin expression may indeed be important for the maintenance of hearing into advanced age.

In summary, we demonstrated that restoration of pendrin to the endolymphatic sac is sufficient to restore normal inner ear function. This implies that pendrin in the endolymphatic sac is more important for the development of normal hearing than pendrin expression in the cochlea and more important for the development of normal balance than pendrin expression in the vestibular labyrinth. This finding, in conjunction with our previous report that pendrin expression is required for embryonic development but not for the maintenance of hearing, opens the prospect that a spatially and temporally limited therapy will restore normal hearing in human patients carrying a variety of mutations of *SLC26A4*.

## Methods

### Ethics statement

All animal experiments and procedures at Kansas State University were performed according to protocols approved by the Animal Care and Use Committees at Kansas State University (IACUC#: 2961). All animal procedures at Sorbonne University Paris Cité were performed according to protocols approved by the ethics committee from University Pierre et Marie Curie, and were performed in accordance with the Guide for the Care and Use of Laboratory Animals (NIH publication No. 93-23, revised 1985).

### Generation of Tg(B1-hPDS)^Tg/+^;Slc26a4^+/+^ transgenic mice

Human *SLC26A4* cDNA was ligated into a pBluescript vector that contained 6.9 kbp of the human *ATP6V1B1* promoter [16], [20]. An SV40 late region polyadenylation signal was cloned downstream of the *SLC26A4* cDNA. The transgene Tg(*B1-hPDS*) included the 5′-flanking region of the *ATP6V1B1* gene extending to but excluding the endogenous translational start codon, the human *SLC26A4* cDNA, with its own translational start site, and the SV40 late region polyadenylation signal. The integrity of the transgene was confirmed by restriction digest and bidirectional sequencing of ligation sites. In preparation for injection, the transgene was linearized by SalI and NotI digestion, followed by gel purification using an electroelution method and then concentrated using ElutipD columns (Whatman). The transgene was then further concentrated by ethanol precipitation and resuspended in low EDTA injection buffer (10 mM Tris with 0.1 mM EDTA). Tg(*B1-hPDS*) transgenic mice were created by the University of Utah transgenic mouse core facility using standard procedures [16], [20]. Genotyping revealed that 63 pups were positive for transgene integration. One founder, which transmitted the transgene in a Mendelian fashion, was crossed with wild-type C57BL/6× CBA F1 mice to establish a colony. Three Tg(*B1-hPDS*);*Slc26a4^+/+^* transgenic mice were shipped to Kansas State University in Manhattan, Kansas, USA.

### Generation of Tg(B1-hPDS)^Tg/+^;Slc26a4^Δ/Δ^ transgenic mice

At Kansas State University, a colony of Tg*(B1-hPDS)* ^Tg/+^;*Slc26a4^Δ/Δ^* mice was established. Colony management was supported by software (Litter tracker, written in Microsoft Visual Basic and Excel 2010 by P.W.) Tg(*B1-hPDS*)^Tg/+^;*Slc26a4^+/+^* mice were crossed with *Slc26a4^Δ/Δ^* mice to generate Tg(*B1-hPDS*)^Tg/+^;*Slc26a4* ^Δ/+^ mice. Matings of Tg(*B1-hPDS*)^Tg/+^;*Slc26a4* ^Δ/+^ mice generated 28 Tg(*B1-hPDS*)^Tg/+^;*Slc26a4* ^+/+^, 58 Tg(*B1-hPDS*)^Tg/+^;*Slc26a4* ^Δ/+^ and 39 Tg(*B1-hPDS*)^Tg/+^;*Slc26a4* ^Δ/Δ^ mice in a near Mendelian ratio of 1 ∶ 2 ∶ 1 with a 75% rate of transmission for the transgene (based on 169 pups).

### Genotyping

Mice were genotyped for *Slc26a4* ^+^ and *Slc26a4* ^Δ^ alleles by PCR using established primers [14] and for the transgene *Tg(B1-hPDS)* (Transnetyx, Cordova, TN). Primers for the transgene were designed to amplify a 345 bp PCR-product spanning the *hPDS* cDNA and the SV40 polyadenylation signal sequence (left primer: 5′-aga ggg tca agg ttc cat ttt ag-3′; right primer: 5′-caa acc aca act aga atg cag tg-3′) [15].

### Isolation of embryonic tissues

Time-pregnant dams were deeply anesthetized with 4% tri-bromo-ethanol (0.014 ml/g body weight, i.p.) and embryos were harvested by laparotomy. Dams and embryos were sacrificed by decapitation. Gestational age was counted from the day when the vaginal plug was detected. This day was set to embryonic (E) day 0.5. Gestational age, however, was verified by evaluating gross morphological features including limbs, digits and the appearance of the pinna and auditory meatus [36], [37].

### Isolation of tissues from postnatal mice

The age of mice was counted from the day of birth, which was set to postnatal (P) day 0. Postnatal mice were deeply anesthetized with 4% tri-bromo-ethanol (0.014 ml/g body weight, i.p.) and sacrificed by decapitation or cardiac perfusion with fixative.

### Quantitative RT-PCR

Quantitative RT-PCR was performed on total RNA [17]. Total RNA was isolated from tissues obtained by microdissection from Tg(−);*Slc26a4* ^Δ/+^ and Tg(+);*Slc26a4* ^Δ/Δ^ mice and subjected to quantitative RT-PCR using gene-specific primers for 18S rRNA as well as for mRNA coding for the α-subunit of the mouse Na^+^/K^+^ ATPase *Atp1a1*, the B1-subunit of the mouse vH^+^ATPase *Atp6v1b1*, mouse pendrin *Slc26a4* and human pendrin *SLC26A4*, which was introduced via the transgene.

Postnatal mice were genotyped by PCR prior to tissue collection. Embryonic Tg(−);*Slc26a4* ^Δ/+^ mice were generated by mating Tg(−);*Slc26a4* ^Δ/Δ^ dams and Tg(−);*Slc26a4* ^+/+^ sires, which yielded 100% of the desired genotype. Embryonic Tg(+);*Slc26a4* ^Δ/Δ^ mice were generated by mating Tg(+);*Slc26a4* ^Δ/Δ^ dams and sires, which yielded Tg(+);*Slc26a4* ^Δ/Δ^ and Tg(−);*Slc26a4* ^Δ/Δ^ mice in a ratio of 3 ∶ 1. Since embryonic mice could not be genotyped prior to tissue collection, the desired Tg(+);*Slc26a4* ^Δ/Δ^ mice among Tg(−);*Slc26a4* ^Δ/Δ^ mice were initially identified by visual inspection of the size of the endolymphatic sac and the presence of ‘glittering’ otoconia, This phenotypic identification was subsequently confirmed by the presence of human pendrin *SLC26A4* transgene by RT-PCR.

Tissues were obtained by microdissection. Endolymphatic sacs (8–10 endolymphatic sacs from 4–5 animals per sample) were obtained from mice at age E17.5. Cochlear ducts (4 cochlear ducts from 2 animals per sample and 2 cochlear ducts from 1 animal per sample) were obtained from mice at ages E17.5 and P2, respectively. Vestibular labyrinths (6 vestibular labyrinths from 3 animals per sample) were obtained from mice at age P8. Total RNA was isolated from microdissected tissues (RNeasy micro kit, Qiagen, Valencia, CA, USA), treated with DNAse (RNeasy micro kit), combined with RNA storage solution (Applied Biosystems/Ambion, Austin, TX), adjusted to a concentration of 10 ng/µl, and stored at −80°C.

Quantity and quality of total RNA were evaluated by microfluidic electrophoresis (BioAnalyzer, Agilent, Santa Clara, CA), by microliter absorption photometry (Nanodrop, Wilmington, DE) and by quantitative RT-PCR of 18S rRNA. RNA samples were accepted for quantitative RT-PCR only when they were free of contamination and excellent RNA quality. RNA quality was quantified by the RNA integrity number (RIN) on a scale from 0 (worst) to 10 (best) (BioAnalyzer, Agilent). RIN numbers for total RNA isolated from E17.5 endolymphatic sac and cochlea were 8.2±0.3 (n = 3) and 9.2±0.1 (n = 10).

Chemicals were assembled with the assistance of an automatic pipetting station (Biomek NX^p^, Beckman Coulter, Fullerton, CA) with hardware modifications and software programming by P.W. Quantitative RT-PCR reactions were carried out in 96-well plates with each well containing ∼10 ng of total RNA, gene specific primers, and an enzyme mix containing reverse transcriptase and DNA polymerase (iScript, BioRad, Hercules, CA) in a total volume of 25 µl. Reverse transcription was performed for 10 min at 50°C and terminated by heating to 95°C for 5 min (OneStepPlus, Applied Biosystems, Foster City, CA). PCR consisted of 40 cycles of 10 s melting at 95°C, 30 s annealing and elongation at 58°C, and 15 s hot-measurement at 78°C (OneStepPlus, Applied Biosystems).

Left and right primers (exon, product size) were for 18S 5′-gag gtt cga aga cga tca ga-3′ and 5′-tcg ctc cac caa cta aga ac-3′ (316 bp), for *Atp1a1* 5′-tgc ccg cct caa cat tcc-3′ (exon 14) and 5′-gac aca tca gag cca aca atc c-3′ (exon 16, 291 bp), for *Atp6v1b1* 5′-tga ccc gaa act aca tca cc-3′ (exon 1) and 5′-gcc aga gcc att gaa aat cc-3′ (exon 5, 305 bp), for mouse *Slc26a4* 5′-tct gat gga ggc aga gat ga-3′ (exon 20) and 5′-ggc cag cct aac aga gac ag-3′ (exon 21, 430 bp), and for human *SLC26A4* were 5′-tcc caa agt gcc aat cca ta-3′ and 5′-aca tca agt tct tct tcc gtc ag-3′ (360 bp). Primer pairs for *Atp1a1*, *Atp6v1b1*, mouse *Slc26a4* spanned introns to prevent amplification of genomic DNA. Primer pairs for mouse *Slc26a4* detected the *Slc26a4^+^* allele as well as the *Slc26a4^Δ^* allele that is lacking exon 8 [14]. Left and right primers for mouse *Slc26a4* differed by 7 and 10 nucleotides from the corresponding human sequence and left and right primers for human *SLC26A4* differed in 4 and 6 nucleotides from the corresponding mouse sequence, thereby maximizing species-specific amplification. Since the human transgene did not contain introns, some reactions were carried out without reverse transcriptase to determine whether products of *SLC26A4* originated from cDNA rather than from genomic DNA. These experiments revealed no evidence for significant amplification of genomic DNA. Amplification of a single product of the appropriate size was verified by microfluidic electrophoresis (BioAnalyzer, Agilent).

The number of template molecules (*cDNA_Template_*) was estimated according to
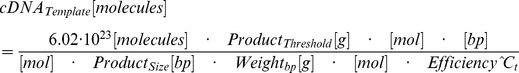
where 6.02×10^23^ molecules/mol represents Avogadro's number, *Product_Threshold_* is the weight of the PCR-product at threshold (0.49×10^−9^ g) that was obtained from calibration experiments, *Product_Size_* is the size of the product in base pairs (bp), *Weight_bp_* is average weight of one bp (660 g/mol), *Efficiency* is the PCR-efficiency obtained from the slope of the log-linear phase of the growth curve [38] and *C_t_* is the cycle at which the fluorescence of the product molecules reaches a common threshold chosen in the middle of the log-linear part of the growth curve.

### Paint-fill

Bisected heads of embryos age E15.5 were fixed overnight in Bodian's fixative, contained (vol/vol) 75% ethanol, 5% acetic acid, and 5% formalin in water. Heads were then dehydrated overnight in 100% ethanol and cleared in methyl-salicylate [39], [40]. The membranous labyrinth was injected via the lateral wall of the basal turn of the cochlea and via the endolymphatic sac with diluted paint (Liquid Paper, Newell Rubbermaid, Atlanta, GA, 0.1–0.2% in methyl-salicylate) using a fine glass-electrode, a manipulator (NM-151 Narishige) and a micrometer-driven oil-filled microinjector (CellTram Vario, Eppendorf, Hamburg, Germany). For each genotype, at least three inner ears were injected.

### Direct fluorescence of eGFP

Whole mounts of fresh cochlear ducts, endolymphatic sacs and slices of kidney were prepared from E15.5 Tg(*B1-eGFP*) mice and visualized with a fluorescence microscope (AxioScope, Carl Zeiss Göttingen).

### Western blotting

#### Crude membrane preparations of the inner ear

Soft tissues from 8 cochleae and 8 vestibular labyrinths devoid of endolymphatic sacs from adult mice were collected by microdissection in Cl^−^-free solution and pooled and homogenized in 500 µl Tris-sucrose buffer. Cl^−^-free solution contained (mM) 150 Na-gluconate, 1.6 K_2_HPO_4_, 0.4 KH_2_PO_4_, 4 Ca-gluconate_2_, 1 MgSO_4_ and 5 glucose, pH 7.4. Tris-sucrose buffer contained (mM) 250 sucrose, 50 Tris-HCl, 5 EDTA, pH = 7.4, and proteinase inhibitor cocktail (Cat# PI-87786 Fisher, Pittsburgh, PA). Homogenization involved a pestle/1.5 ml Eppendorf vial and a Potter-Elvehjem tissue grinder (3 strokes at 1,250 rpm; Cat# K885512-0020, Fisher). Nuclei and debris were sedimented by centrifugation (6 min, 600 g, 4°C, Micromax RF, International equipment, Needham Heights, MA) and the supernatant containing membranes was collected. Pellets were washed 3× with 100 µl Tris-sucrose buffer. Supernatants were pooled and membranes were pelleted by centrifugation (1 hr, 21,000 g, 4°C, Micromax RF). Membranes were suspended in 30 µl Tris-sucrose buffer and protein content was quantified (BCA assay, Cat# 23227, Fisher; Absorption photometer, Nanodrop).

#### Crude membrane preparations of kidneys

Kidneys (∼1 g wet weight) were sliced, frozen in liquid N_2_, pulverized, transferred into 8 ml Tris-sucrose buffer and homogenized. Homogenization involved pestles fitting 1.5 ml Eppendorf vials and the Potter-Elvehjem tissue grinder (3 strokes at 1,250 rpm). Nuclei and debris were sedimented by centrifugation (6 min, 600 g, 4°C, Micromax RF) and the supernatant containing membranes was collected. Pellets were washed 3× with 2 ml Tris-sucrose buffer. Supernatants were pooled and membranes were pelleted by centrifugation (1 hr, 21,000 g, 4°C, Micromax RF). Membranes were suspended in 200 µl Tris-sucrose buffer and protein content was quantified (BCA assay).

#### Gel-electrophoresis, blotting and protein detection

Crude membranes were solubilized, denatured and reduced for 1 hr at 37°C in LDS sample buffer (Cat#: NP0007; Invitrogen) supplemented with 50 mM DTT. Proteins were resolved by gel-electrophoresis (4–12% Bis-Tris gels (Cat#: NP0326, Invitrogen) using a MOPS-SDS running buffer supplemented with an antioxidant (Cat#: NP0005, Invitrogen) and transferred onto PVDF membranes (BioRad, 3 hrs at 30 V). PVDF membranes were blocked for 1 hr at RT in TBST solution containing 3% BSA. TBST solution contained 20 mM Tris-base, 68 mM NaCl, and 1% Tween-20. Blocked PVDF membranes were incubated overnight at 4°C with primary antibody in TBST solution with 3% BSA. Primary antibodies were a rabbit anti-pendrin antibody (Pds #2; 1∶200) and a polyclonal rabbit anti-mouse β-actin antibody (1∶ 1,000; Cat#: A2066, Sigma-Aldrich). Pds #2 was raised against the last 15 amino acids of the C-terminal of mouse pendrin [30]. Human pendrin differs in only 1 of these 15 amino acids. PVDF membranes were washed 5×3 min in TBST solution and then incubated for 1 hr at RT with the secondary antibody, goat anti-rabbit poly-HRP (Cat#: PI32260, Fisher) diluted in TBST solution with 3% BSA. The dilution of the secondary antibody was 1∶2,500 for labeling the Pds #2 antibody and 1∶10,000 for labeling the β-actin antibody. The membranes were washed 3×5 min in TBST solution. HRP was detected by chemiluminescence (SuperSignal Femto, Cat# PI34095, Fisher). The signal was integrated over 2–5 min by cooled CCD camera (ImageStation 4000R, Kodak).

### Immunocytochemistry

#### Fixation

Isolated tissues from embryonic mice were fixed by submersion in PBS-formaldehyde solution at 4°C. PBS-formaldehyde solution consisted of 4% formaldehyde (Electron Microscopy Sciences, Hatfield PA) in a solution that contained (in mM) 137 NaCl, 2.7 KCl, 10.1 Na_2_HPO_4_, 1.8 KH_2_PO_4_. Tissues from postnatal animals were fixed by cardiac perfusion followed by perilymphatic perfusion. Cardiac perfusion was initiated with Cl^−^-free solution (6 ml, 1 min) and followed by Cl^−^-free solution with 4% formaldehyde (12 ml, 2 min). Perilymphatic perfusion consisted of an infusion of Cl^−^-free solution with 4% formaldehyde (25 µl, 5 min) through the round and oval window of the cochlea.

#### Cryosections

Fixed temporal bones were decalcified 18–24 h in 10% EDTA, processed through a sucrose gradient and infiltrated with polyethylene glycol. Mid-modiolar cryosections (12 µm, CM3050S, Leica, Nussloch, Germany) were blocked in PBS-TX (137 mM NaCl, 10.1 mM Na_2_HPO_4_, 1.8 mM KH_2_PO_4_, 2.7 mM KCl, pH 7.4 with 0.15% Triton X 100) and 5% bovine serum albumin. Slides were incubated overnight at 4°C with primary antibody in PBS-TX with 2.5% BSA. Primary antibodies were two rabbit anti-pendrin antibodies (Pds #1; 1∶200; a kind gift from Dr. Søren Nielsen, University of Aarhus, Denmark and Pds #2, 1∶1,000) and a polyclonal FITC-conjugated goat anti-eGFP antibody (Cat#: GTX26662, GeneTex, San Antonio, TX). Pds #1 was raised against the last 22 amino acids of the C-terminal of mouse pendrin. Human pendrin differs in 6 of these 22 amino acids [13], [41]. Slides were washed with PBS-TX and, where appropriate, incubated for 1 h at RT with secondary antibody (1∶1,000, goat anti-rabbit Alexa 594, Invitrogen, Carlsbad, CA). After washing with PBS-TX, sections, where appropriate, were stained with phalloidin 488 (1∶40; Invitrogen) to mark F-actin and DAPI (1∶1,000; Invitrogen) to mark nuclei, washed again and cover-slipped with mounting medium (Vectashield HardSet Mounting Medium, Vector laboratories, Burlingame, CA).

#### Whole-mounted specimens

Tissues were obtained by microdissection from fixed temporal bones, washed in PBS-TX, and blocked with 5% bovine serum albumin in PBS-TX. Tissues were incubated overnight at 4°C with primary antibodies in PBS-TX with 2.5% BSA. Primary antibodies were a rabbit anti-pendrin antibody (Pds #1; 1∶200) or a polyclonal FITC-conjugated goat anti-eGFP antibody (GeneTex). Slides were washed with PBS-TX and, where appropriate, incubated for 1 h at RT with secondary antibody (1∶1,000, goat anti-rabbit Alexa 594, Invitrogen). After washing with PBS-TX, tissues were stained with phalloidin 488 or phalloidin 594 (1∶40; Invitrogen) and DAPI (1∶1000; Invitrogen), washed again and cover-slipped with mounting medium (VectaShield HardSet Mounting Medium, Vector laboratories, Burlingame, CA).

#### Confocal microscopy

Immunocytochemistry of cryosections and whole-mounted specimens were viewed by confocal microscopy (LSM 510 Meta, Carl Zeiss, Göttingen, Germany). Laser scanning bright-field images were collected to aid orientation.

### Endocochlear potential and pH difference between endolymph and perilymph

Mice were anesthetized with 4% tri-bromo-ethanol for *in situ* measurements of the endocochlear potential and pH with double-barreled microelectrodes. Measurements were made in the basal turn of the cochlea by a round-window approach through the basilar membrane of the first turn of the cochlea [18], [42]. The surgical cavity was covered with liquid Sylgard 184 (Dow Corning) to limit the loss of tissue CO_2_ into ambient air.

Double-barreled glass microelectrodes were pulled (micropipette puller PD-5; Narishige) from filament-containing glass tubing (1B100F-4; World Precision Instruments) and baked at 180°C for 2 h to ensure dryness. One barrel was silanized by a 30 s exposure to 0.008 ml dimethyldichlorosilane (40136; Fluka). After silanization, microelectrodes were baked again at 180°C for 3 h and tips were broken to a final O.D. of ∼3 µm. The reference barrel was filled with 150 mM KCl and the ion-selective barrel was filled at the tip with liquid ion exchanger (Hydrogen ionophore II - Cocktail A, 95297; Fluka) and back-filled with buffer solution (500 mM KCl, 20 mM HEPES, pH 7.4).

Each barrel of the double-barreled microelectrode was connected via a Ag/AgCl_2_ electrode to an electrometer (FD223, World Precision Instruments). A flowing KCl electrode (1 M KCl in 0.2% agar) was inserted under the skin of the animal to serve as ground electrode. Data were recorded in analog (BD12E Flatbed recorder, Kipp & Zonen, Delft, The Netherlands) and digital form (DIGIDATA 1322A and AxoScope 10, Axon Instruments, Union City, CA). pH electrodes were calibrated *in situ* at 37°C using three calibration solutions with different pH values. Calibration solutions contained (in mM): pH 6: 130 NaCl, 20 MES; pH 7: 130 NaCl, 20 HEPES; and pH 8: 130 NaCl, 20 HEPES. pH-sensitive electrodes had a slope of 56.9 ± 0.3 mV/pH unit (n = 11).

### Hearing tests

Mice were deeply anesthetized with a mixture of dexmedetomidine and ketamine (0.375 mg/Kg body weight dexmedetomidine and 56 mg/kg body weight ketamine; i.p.) and placed on a thermal pad to maintain normal body temperature. The mastoid, vertex and ventral neck region of the animal were connected via sub-dermal platinum needle electrodes (F-E2, Astro Med, Rhode Island, RI) and short (31 cm) leads to the main channel, reference channel and ground of the preamplifier, respectively. Auditory brainstem recordings were performed in a custom constructed, electrically shielded and sound-attenuated chamber (inner dimensions: 23 cm×23 cm×23 cm) using a digital data acquisition system (BioSig32 software, RA4LI Preamplifier, RP2.1 Enhanced Real Time Processor, PA5 Programmable Attenuator, ED1 Electrostatic Speaker Driver, Tucker-Davis Technologies, Alachua, FL). Tone burst stimuli were presented (21 per sec) via a free field electrostatic speaker (SigGen software, ES1 speaker, Tucker Davis). Acoustic stimuli were calibrated using a 1/4 inch condenser microphone (SigCal IRP4.2 software, Tucker Davis, PS9200 microphone, Acoustical Interface, Belmont, CA) placed at the location of the mouse head. Tone bursts (2 ms duration, 0.5 ms gate time; 8, 16 and 32 kHz) were presented with alternating phase (0 and 180°). Responses, recorded over 10 ms, were filtered (300 Hz high pass, 3000 Hz low pass and 60 Hz notch) and 1000 recordings were averaged. Tone burst stimuli were presented at intensities varying between 90 and 0 dB SPL in 5 dB intervals. Auditory thresholds were obtained by a visual comparison of wave forms. After the procedure, mice were rapidly recovered from anesthesia with atipamizole (1.875 mg/kg body weight; i.p.).

### Balance tests

Balance testing consisted of determining the time that mice could balance on a rotating 1″ rod with rotations ramping up from 4 to 40 rpm in 60 s (RotaRod, IITC Life Science, Woodland Hills, CA). Test chambers were cushioned with bubble-foil to provide a soft landing for mice falling off the rod.

## Supporting Information

Figure S1 Histology and pendrin expression in the cochlea. Staining in all images consisted of immunocytochemistry of pendrin (Pds #1 antibody or Pds #2 antibody; *red*), F-actin (*green*) and nucleic acids (*blue*). Images provide comparison of pendrin expression in Tg(+);*Slc26a4* ^Δ/Δ^ mice (**A, C, E**) to positive controls consisting of Tg(+);*Slc26a4* ^Δ/+^ (**B, D**) or Tg(−);*Slc26a4* ^Δ/+^ mice (**F**). Note that both antibodies, Pds #1 and Pds #2, failed to detect pendrin expression in Tg(+);*Slc26a4* ^Δ/Δ^ mice and that the staining pattern for pendrin in positive controls was similar for both antibodies and for both positive controls. The number of pairs of mice represented by these images are 2 for images **A** & **B**, 1 for **C** & **D**, and 2 for **E** & **F** with 3 sections being evaluated per animal. K, Kölliker's organ; OS, outer sulcus; Lim, spiral limbus; IS, inner sulcus; OC, organ of Corti; SP, spiral prominence; SV, stria vascularis; RM, Reissner's membrane. Compare these images to those in Fig. 7.
(TIFF)

Figure S2 Histology and pendrin expression in the vestibular labyrinth. Staining in all images consisted of immunocytochemistry of pendrin (Pds #1 antibody or Pds #2 antibody; *red*) and F-actin (*green*) and of nucleic acids (*blue*). Images provide comparison of pendrin expression in Tg(+);*Slc26a4* ^Δ/Δ^ mice (**A,C,E,G,I,K**) to positive controls consisting of Tg(+);*Slc26a4* ^Δ/+^ (**B, F, J**) or Tg(−);*Slc26a4* ^Δ/+^ mice (**D,H,L**). Note that both antibodies, Pds #1 and Pds #2, failed to detect pendrin expression in Tg(+);*Slc26a4* ^Δ/Δ^ mice and that the staining pattern for pendrin in positive controls was similar for both antibodies and for both positive controls. The number of pairs of mice represented by these images are 2 for images **A** & **B**, 2 for images **C** & **D**, 1 for images **E** & **F**, 2 for images **G** & **H**, 2 for images **I** & **J**, and 2 for images **K** & **L**. HC, vestibular hair cells; TC, transitional cells; M, melanocytes. Compare these images to those in Fig. 10.
(TIFF)
