# Supplementary figures and images for: SLC26A4 Targeted to the Endolymphatic Sac Rescues Hearing and Balance in Slc26a4 Mutant Mice
Source: PLoS Genet. 2013 Jul 11;9(7):e1003641. doi: 10.1371/journal.pgen.1003641 (PMC3708829; doi:10.1371/journal.pgen.1003641)

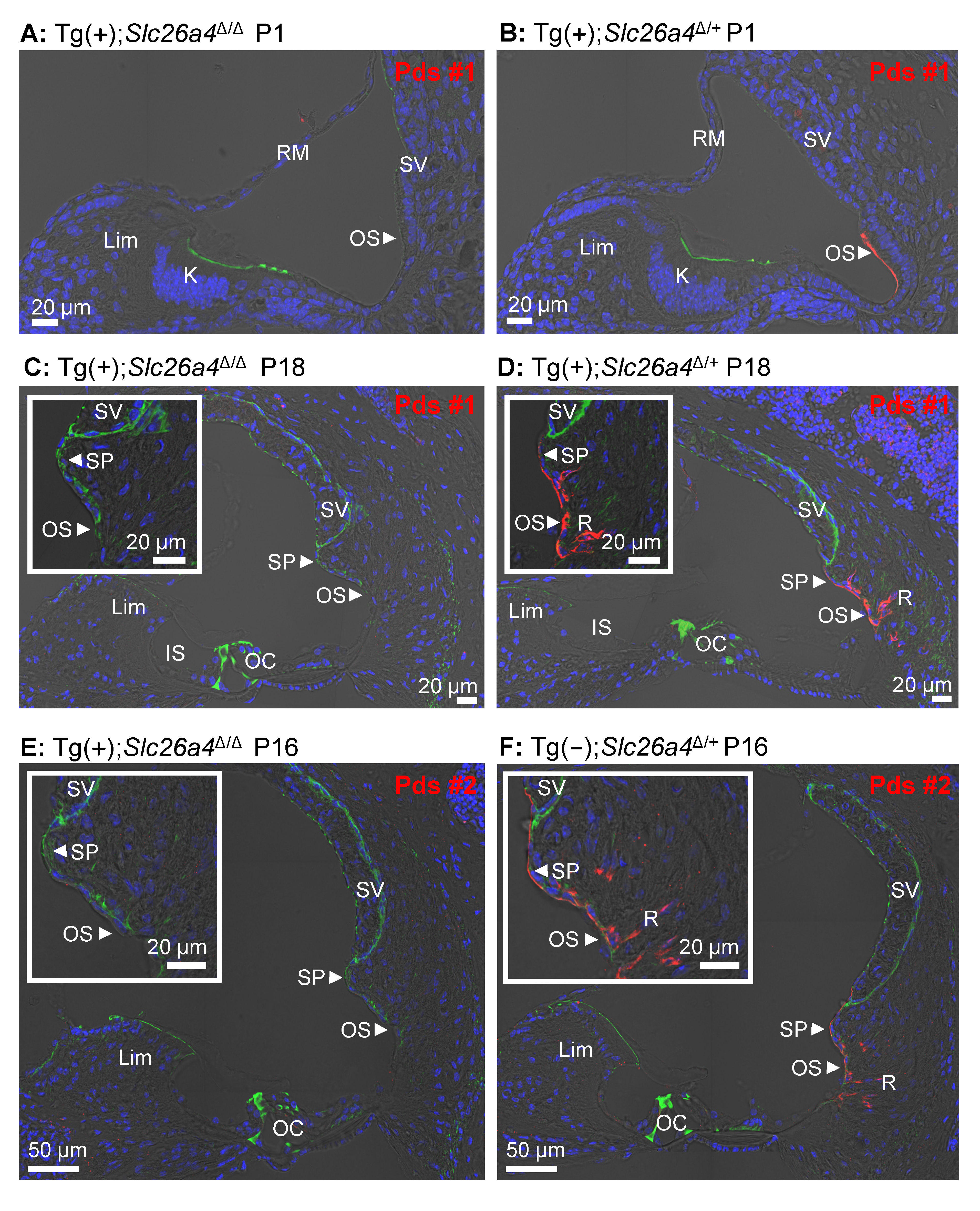

Supplement: Figure S1 — Histology and pendrin expression in the cochlea. Staining in all images consisted of immunocytochemistry of pendrin (Pds #1 antibody or Pds #2 antibody; red), F-actin (green) and nucleic acids (blue). Images provide comparison of pendrin expression in Tg(+);Slc26a4 Δ/Δ mice (A, C, E) to positive controls consisting of Tg(+);Slc26a4 Δ/+ (B, D) or Tg(−);Slc26a4 Δ/+ mice (F). Note that both antibodies, Pds #1 and Pds #2, failed to detect pendrin expression in Tg(+);Slc26a4 Δ/Δ mice and that the staining pattern for pendrin in positive controls was similar for both antibodies and for both positive controls. The number of pairs of mice represented by these images are 2 for images A & B, 1 for C & D, and 2 for E & F with 3 sections being evaluated per animal. K, Kölliker's organ; OS, outer sulcus; Lim, spiral limbus; IS, inner sulcus; OC, organ of Corti; SP, spiral prominence; SV, stria vascularis; RM, Reissner's membrane. Compare these images to those in Fig. 7. (TIFF) [file pgen.1003641.s001.tiff]

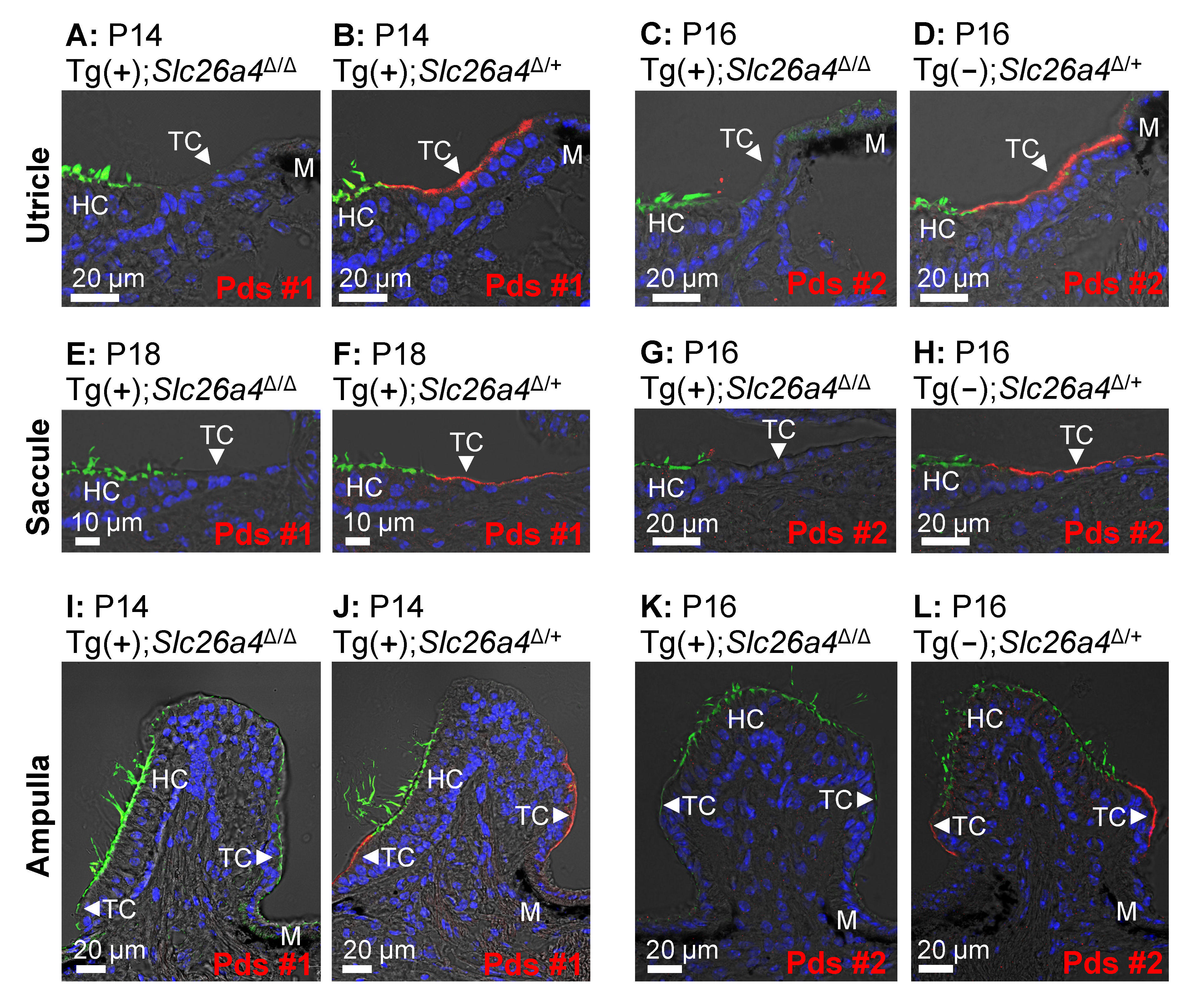

Supplement: Figure S2 — Histology and pendrin expression in the vestibular labyrinth. Staining in all images consisted of immunocytochemistry of pendrin (Pds #1 antibody or Pds #2 antibody; red) and F-actin (green) and of nucleic acids (blue). Images provide comparison of pendrin expression in Tg(+);Slc26a4 Δ/Δ mice (A,C,E,G,I,K) to positive controls consisting of Tg(+);Slc26a4 Δ/+ (B, F, J) or Tg(−);Slc26a4 Δ/+ mice (D,H,L). Note that both antibodies, Pds #1 and Pds #2, failed to detect pendrin expression in Tg(+);Slc26a4 Δ/Δ mice and that the staining pattern for pendrin in positive controls was similar for both antibodies and for both positive controls. The number of pairs of mice represented by these images are 2 for images A & B, 2 for images C & D, 1 for images E & F, 2 for images G & H, 2 for images I & J, and 2 for images K & L. HC, vestibular hair cells; TC, transitional cells; M, melanocytes. Compare these images to those in Fig. 10. (TIFF) [file pgen.1003641.s002.tiff]
